# Placental epigenetics for evaluation of fetal congenital heart defects: Ventricular Septal Defect (VSD)

**DOI:** 10.1371/journal.pone.0200229

**Published:** 2019-03-21

**Authors:** Uppala Radhakrishna, Samet Albayrak, Rita Zafra, Alosh Baraa, Sangeetha Vishweswaraiah, Avinash M. Veerappa, Deepthi Mahishi, Nazia Saiyed, Nitish K. Mishra, Chittibabu Guda, Rouba Ali-Fehmi, Ray O. Bahado-Singh

**Affiliations:** 1 Department of Obstetrics and Gynecology, Oakland University William Beaumont School of Medicine, Royal Oak, Michigan, United States of America; 2 Department of Obstetrics and Gynaecology, Wayne State University School of Medicine, Detroit, Michigan, United States of America; 3 Department of Obstetrics and Gynaecology, Icahn School of Medicine at Mount Sinai, New York, New York, United States of America; 4 Department of Pathology, Wayne State University School of Medicine, Detroit, Michigan, United States of America; 5 Department of Studies in Genetics and Genomics, Laboratory of Genomic Sciences, University of Mysore, Mysore, India; 6 Biotechnology, Nirma Institute of Science, Nirma University, Ahmedabad, India; 7 Department of Genetics, Cell Biology & Anatomy, College of Medicine, University of Nebraska Medical Centre Omaha, Nebraska, United States of America; UNC Eshelman School of Pharmacy, UNITED STATES

## Abstract

Ventricular Septal Defect (VSD), the most common congenital heart defect, is characterized by a hole in the septum between the right and left ventricles. The pathogenesis of VSD is unknown in most clinical cases. There is a paucity of data relevant to epigenetic changes in VSD. The placenta is a fetal tissue crucial in cardiac development and a potentially useful surrogate for evaluating the development of heart tissue. To understand epigenetic mechanisms that may play a role in the development of VSD, genome-wide DNA methylation assay on placentas of 8 term subjects with isolated VSD and no known or suspected genetic syndromes and 10 unaffected controls was performed using the Illumina HumanMethylation450 BeadChip assay. We identified a total of 80 highly accurate potential CpGs in 80 genes for detection of VSD; area under the receiver operating characteristic curve (AUC ROC) 1.0 with significant 95% CI (FDR) p-values < 0.05 for each individual locus. The biological processes and functions for many of these differentially methylated genes are previously known to be associated with heart development or disease, including cardiac ventricle development (*HEY2*, *ISL1*), heart looping (SRF), cardiac muscle cell differentiation (*ACTC1*, *HEY2*), cardiac septum development (ISL1), heart morphogenesis (*SRF*, *HEY2*, *ISL1*, *HEYL*), Notch signaling pathway (*HEY2*, *HEYL*), cardiac chamber development (*ISL1*), and cardiac muscle tissue development (*ACTC1*, *ISL1*). In addition, we identified 8 microRNAs that have the potential to be biomarkers for the detection of VSD including: miR-191, miR-548F1, miR-148A, miR-423, miR-92B, miR-611, miR-2110, and miR-548H4. To our knowledge this is the first report in which placental analysis has been used for determining the pathogenesis of and predicting VSD.

## Introduction

Congenital Heart Defect (CHD) affects nearly 40,000 newborns per year in the United States [1,2]. Ventricular Septal Defect (VSD) is the most common congenital heart disease (CHD) and occurs in approximately 1 in 500 live births [3–5]. The frequency is even more common in prenatal life. Common risk factors for VSD include family history, ethnicity, and a few genetic disorders. Data indicate that genetic mutations of cardiac developmental genes such as *NKX2-5*, *SMAD3*, *NTRK3*, *GATA6*, *TBX2*, *TBX18*, *ATA6*, *and TBX2*, unique copy number variations [6], and chromosomal aneuploidy such as trisomy 13, 18, or 21 can play an important role in the etiology of VSD [7]. Targeted disruption of the CHF1/Hey2 locus in mice resulting in VSD has also been reported [8]. Despite these known genetic disruptions, in the clinical setting the cause of VSD remains unknown in the great majority of cases.

The heart and the placenta are the two first organs to develop in pregnancy. Their development is inter-related, a phenomenon known as the ‘placental-heart’ axis. This relationship is not widely understood, one consequence of which has been insufficient studies in humans to determine how early placental function contributes to the downstream development of CHD. Multiple lines of evidence: genetic [9], hemodynamic and vascular [10], metabolic, eg. folate deficiency [9], and others [11], do suggest a significant potential role of the placenta in the development of non-syndromic CHD.

DNA methylation is one of multiple different but interrelated epigenetic mechanisms and is currently the most widely studied. DNA methylation is a significant player in the transcriptional regulation of genes and aberrant methylation has been implicated in many complex and common diseases including cancer, diabetes, and psychiatric disorders. While insufficiently studied, emerging evidence suggests that DNA methylation may have an important role in both normal and pathologic heart development [12]. Methylation of CpG islands is classically thought to have its biological effect by displacing transcription binding factors, and instead attracting methyl binding factors that condense DNA and suppress gene expression. Environmental factors such as maternal diet, smoking and alcohol exposure profoundly affect DNA methylation. These factors are known to be risk modifiers for CHD development [1,13,14]. Further, DNA methylation changes have been demonstrated in the cardiac tissue DNA of CHD fetuses [15].

While methylation is known to be tissue-specific it is not tissue-exclusive. Several recent studies have shown correlation between CpG methylation-specific cytosine loci across different tissues [16,17]. This has raised the prospect of using the DNA methylation status in easily accessible tissues, such as blood leucocytes, to evaluate methylation status and by inference gene activity and disease status in inaccessible organs such as the brain [18]. Epigenetic markers in blood leucocytes have thus been used to detect psychiatric disorders such as schizophrenia [19]. The degree of correlation in cytosine methylation across different tissues is thought to vary based on the specific tissues involved.

Currently, there are no biomarkers available in clinical practice for the pre- or post-natal detection of congenital heart defects (CHDs). Given the significance of CHD and the frequency of missed or late diagnosis, this is a major deficiency. In our prior pilot data [20,21] cytosine methylation status of blood leucocytes was found to be a potentially useful molecular biomarker for the detection of multiple different categories of CHD. This is consistent with attempts to use epigenetic signatures in leucocytes to detect psychiatric disorders [19]. The placenta, like newborn blood that was used in our prior studies, is a fetal tissue. We therefore reasoned that although not identical, there was likely to be a significant minority of cytosine loci in which parallel or correlated epigenetic modifications could be identified in cardiac development genes in general and specific genes related to ventricular development in the placenta from VSD pregnancies. Our objective therefore in this proof-of-concept study was to evaluate the utility of determining cytosine methylation patterns in placental DNA to elucidate the pathogenesis of and for the detection of isolated non-syndromic VSD. Further, we used cytosine methylation to investigate the molecular pathogenesis of non-syndromic VSD based on the associated genes and gene pathways that were differentially methylated in the VSD cases.

MicroRNA (miRNA) is another important epigenetic mechanism that exerts control over DNA methylation and suppresses gene expression, among other functions. Recent data suggest an important role for miRNA in CHD development [22]. We therefore also evaluated methylation status of known microRNA genes in lieu of measuring actual miRNA levels. Given that DNA methylation status is known to correlate with gene expression, this approach can be used to identify miRNAs that are involved in cardiac development and thus further elucidate the mechanism of VSD pathogenesis. To our knowledge, there are no prior reports using placental molecular analysis for the detection of investigation of CHD pathogenesis.

## Materials and methods

IRB approval to evaluate placental tissue and review maternal and pediatric medical records was provided by Wayne State University. The need to obtain patient consent was waived. CHD was diagnosed based on newborn echo, with cardiac catheterization as clinically dictated. Known or suspected genetic syndromic cases were excluded from the study.

### Tissue samples for analysis

Genomic DNA was isolated from archived formalin-fixed, paraffin-embedded (FFPE) tissue blocks using Puregene DNA Purification kits (Gentra systems MN, USA) according to manufacturer’s protocol. The tissue samples were taken from the placenta of VSD subjects immediately after birth. DNA from archived paraffin-embedded tissue is a suitable template and has been used previously for genome-wide DNA methylation profiles using the Infinium HumanMethylation450 BeadChip assay. Importantly, it has been demonstrated that it is possible to achieve high-performance outcomes using FFPE-derived DNA in genome mapping arrays [23]. FFPE-derived DNA captures on average more than 99% of the CpG sites on the array.

### Genome-wide methylation analysis using the HumanMethylation450

The HumanMethylation450 BeadChip (Illumina, Inc., California, USA) contains >485,000 CpGs per sample in enhancer regions, gene bodies, promoters and CpG islands at a single-nucleotide resolution and requires only 500 ng of genomic DNA. Many studies have previously used Illumina Infinium technology to assess DNA Methylation changes in the placenta associated within fetal disorders [24]. DNA was bisulfite converted using the EZ DNA Methylation-Direct Kit (Zymo Research, Orange, CA), and fluorescently-stained BeadChips imaged by the Illumina iScan. Data were analyzed with Illumina’s Genome Studio methylation analysis package program. The methodology has been previously detailed earlier [20].

As previously reported, to avoid potential confounding factors, probes associated with sex chromosomes and/or containing SNPs in the probe sequence (listing dbSNP entries near or within the probe sequence, i.e., within 10 bp of the CpG site) were excluded from further analysis [25–27]. Probes targeting CpG loci associated with SNPs near or within the probe sequence may influence corresponding methylated probes [28]. The remaining CpG sites were analyzed.

### Statistical and bioinformatic analysis

Differential methylation was assessed by comparing the ß-values per individual nucleotide at each CpG site between VSD subjects and controls. The p-value for methylation differences between case and normal groups at each locus was calculated [29]. Filtering criteria for p-values was set at <0.05 and <0.01 to identify the most discriminating cytosines or the most important differentially methylated regions of the genome. P-values were calculated with False Discovery Rate (FDR) correction for multiple testing (Benjamini-Hochberg test). Further analysis of the differentially methylated genes was conducted for potential biological significance. Receiver Operating Characteristic (ROC) Area Under Curve (AUC) was calculated with R to determine the diagnostic accuracy of specific cytosine loci differentiating CHD from control groups. Data were normalized using the Controls Normalization Method.

The most significantly differentially methylated CpG sites were selected based on pre-set cutoff criteria of ≥2.0-fold increase or ≥2.0-fold decrease with Benjamini -Hochberg FDR p < 0.01. Multiple CpG sites within a gene were resolved by selection of the CpG with the highest fold-change ranking and the lowest FDR p-value. Fold changes in methylation variation were obtained by dividing the mean ß-value for the probes in each CpG site in cases by that of the normal controls. After this filtering, a threshold was set to select ROC curves based on sensitivity plotted against specificity using different ß-value threshold at each CPG locus for VSD prediction, and the corresponding AUC (95% CI) was calculated. In the case of multiple differentially methylated cytosine loci in the same gene we used the locus with the highest discriminating power as defined. Thus only one CpG locus per gene was considered. To avoid potential experimental confounding, various statistical modeling approaches were used. Markers with AUC ≥0.80 and significant 95% CI and FDR p-value < 0.005, i.e., markers with good diagnostic accuracy, were further used to generate heatmap and pathway analysis.

### Gene ontology analysis and functional enrichment

Pathway analysis was carried out using Ingenuity Pathway Analysis (Ingenuity Systems, www.ingenuity.com) and Wikipathways (https://www.wikipathways.org/index.php/WikiPathways) using differentially methylated genes at FDR p-value < 0.01. Literature data mining for co-occurrence of gene names and keywords of interest was performed using Chilibot (www.chilibot.net). Only genes for which Entrez identifiers were available were used in the Pathway analysis. Over-represented canonical pathways, biological processes and molecular processes were identified. Differentially methylated genes based on FDR p-value, percentage methylation change in VSD cases and controls, and those having an AUC of ≥0.80 were selected for enrichment analysis. Enrichment analysis was conducted with an enrichment evaluation statistical analysis test. The p-values were adjusted with Benjamini & Hochberg correction. Information on this function is available at http://rweb.stat.umn.edu/R/library/stats/html/p.adjust.html. The top 10 pathways with the most significant p values were identified and included in the S1 Table. The categories and Q-values from a False Discovery Rate (FDR)-corrected hyper geometric test for enrichment are reported. Q-values are estimated using the Benjamini-Hochberg procedure with a Q-value cutoff of 0.1.

The identified differentially-methylated genes were used to generate a heatmap using the Complex Heatmap (v1.6.0) R package (v3.2.2). Ward distance was used for the hierarchical clustering of samples [30].

### Logistic regression analysis

To select candidate markers for VSD prediction, logistic regression analysis was performed using a more stringent criterion (FDR *p* ≤5x10^-8^) using MetaboAnalyst 4.0 [31].

### Quantitative pyrosequencing

DNA methylation variations were validated on bisulfite-converted genomic DNA by quantitative pyrosequencing. For validation and verification experiments, bisulfite conversion of 1 μg DNA was performed using the EZ DNA Methylation GoldTM kit (Zymo Research, Cambridge Bioscience, UK). DNA methylation variations were compared with the data obtained from conventional quantitative pyrosequencing.

## Results

Genome-wide DNA methylation analysis of placenta-derived DNA was performed on 8 VSD cases and 10 unaffected controls. Comparison of clinical characteristics between cases and controls are shown in Table 1. There were no significant differences between groups.

**Table 1:** Demographics of VSD cases versus controls.

Parameters	Controls	VSD cases	p-value
N	10	8	
Age weeks (mean ± SD)	29.3 ± 4.9	28.8 ± 6.8	0.88*
GA weeks (mean ± SD)	38w 2/7 ± 1w 4/7	37w 2/7 ± 1w 5/7	0.26*
**Race**			0.81^
• White	7	6	
• AA	3	2	

*t-test

^ Chi-square

### Identification of differentially methylated CpG sites in VSD placenta

**Fig 1:**
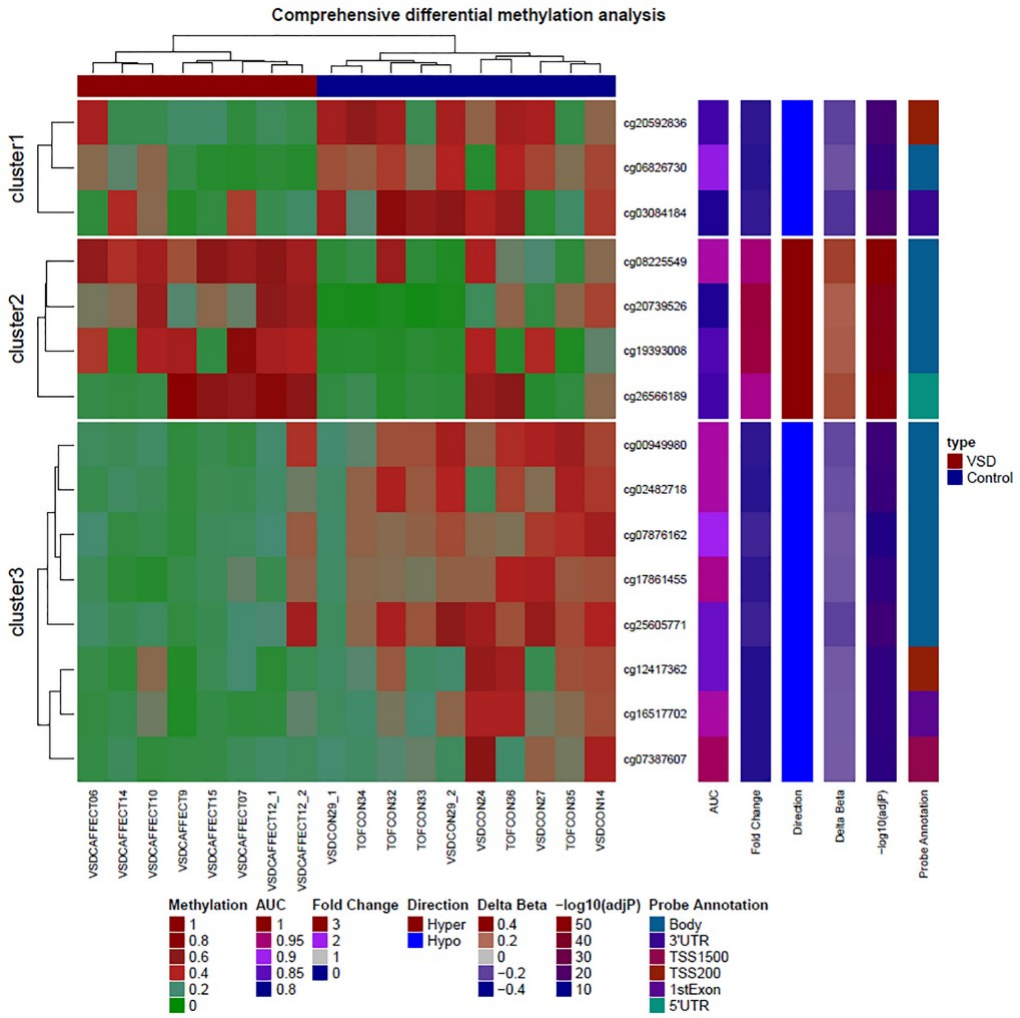
Heatmap of 15 highly differentially methylated loci. Unsupervised hierarchical clustering of differentially methylated loci (rows) in 8 affected and 10 control samples (columns). These 15 CpG loci are at least either 2.0-fold hypomethylated or 2.0-fold hypermethylated with False Detection Rate < 0.00001 in the disease (VSD) condition (red colored squares) compared to normal subjects (blue colored squares). The figure also displays direction, fold change in disease, probe relationship and probe annotation of differentially methylated CpG sites. The red color in the heatmap indicates hyper- DNA-methylation and blue, hypo-DNA-methylation values.

We identified 1328 statistically significant differentially methylated (increased or decreased) CpG regions in 1328 genes in VSD vs control placenta (FDR p-value <0.001). The heat map (Fig 1) visually displays the differential methylation between loci in VSD cases versus controls. Using the most significantly methylated CpG sites as biomarkers for VSD prediction, a total of 80 individual loci (in 80 genes) had an AUC = 1.00 with significant 95% CI for VSD detection. Further, total of 1248 loci (in 1248 genes) had good diagnostic accuracy defined as AUC ≥ 0.81 to 0.99 for VSD detection. In Table 2 we provide the top 55 markers with the stringent p-value<5x10^-8^. The remaining markers will be provided on request to the corresponding author. The ROC curves for four high performing CpG loci with AUC-ROC is depicted in S1 Fig.

**Table 2:** Differentially methylated genes with Target ID, Gene ID, chromosome location, FDR p-value, and % methylation change in VSD cases and controls for each target methylated. CpG sites with significant p-value of 5X10^-8^ indicating methylation status and area under the receiving operator characteristic curve ≥0.80 appear to have strong potential as diagnostic biomarkers for VSD.

Target ID	Gene ID	Chr	FDR p-value	Fold change	% Methylation		AUC
					Cases	Control	
cg08225549	STMN2	8	4.27E-51	2.49	50.55	20.33	0.93
cg26566189	PSORS1C1	6	2.06E-49	2.4	47.65	19.85	0.83
cg19393008	KRT82	12	6.28E-47	2.73	37.73	13.81	0.84
cg20739526	DPP6	7	2.17E-46	2.75	36.18	13.16	0.81
cg03084184	JAKMIP3	10	8.66E-20	0.47	19.45	41.79	0.81
cg20592836	TP53INP2	20	8.82E-18	0.46	17.01	37.27	0.83
cg25605771	SLC6A9	1	1.63E-16	0.49	19.17	39.26	0.86
cg00949980	SUMO3	21	2.43E-16	0.46	16.43	35.53	0.93
cg14001567	TP53TG1; CROT	7	2.39E-15	0.25	4.11	16.66	0.9
cg02482718	AJAP1	1	2.46E-15	0.45	14.46	32.16	0.93
cg06826730	THAP3	1	6.31E-15	0.45	14.13	31.43	0.89
cg17861455	TRPV3	17	5.90E-14	0.46	14.49	31.24	0.94
cg13071729	NAV1	1	6.19E-14	0.32	6.05	18.93	1
cg07068756	UCHL1	4	6.46E-14	0.29	4.98	17.24	0.84
cg04534504	RHOBTB1	10	9.91E-14	0.31	5.48	17.92	0.89
cg01147550	B4GALNT2	17	1.12E-13	0.19	2.55	13.22	0.84
cg07387607	BAT2	6	1.15E-13	0.44	12.27	27.97	1
cg12759024	KLHL32	6	3.71E-13	0.36	7.53	20.72	1
cg07876162	PALM	19	2.43E-12	0.5	16.07	32.2	0.9
cg18478319	SLC15A1	13	3.14E-12	0.3	4.8	15.96	0.88
cg06622999	TRHDE	12	4.68E-12	0.23	3.07	13.13	0.91
cg13944175	HAS1	19	5.91E-12	0.26	3.6	13.91	0.9
cg05852231	ACTC1	15	7.15E-12	0.35	6.18	17.82	0.81
cg10770742	WDR86	7	8.69E-12	0.44	11.06	24.89	0.85
cg20106459	COX6B2	19	6.25E-11	0.28	3.72	13.45	0.86
cg10095938	NKX1-2	10	6.52E-11	0.34	5.39	15.99	0.88
cg14217558	PRDM16	1	1.47E-10	0.37	6.3	17.1	0.88
cg22523852	GABRA5	15	1.48E-10	0.43	8.83	20.78	0.93
cg23944251	GPR158	10	1.50E-10	0.46	10.92	23.73	0.84
cg12405785	HLX	1	1.92E-10	0.22	2.43	11.16	0.88
cg15579587	LRRFIP1	2	3.59E-10	0.29	3.79	13.08	0.9
cg18496317	PRIC285	20	6.32E-10	0.28	3.39	12.31	0.9
cg06848047	SNCA	4	7.46E-10	0.48	11.28	23.65	0.88
cg13124863	SCARA5	8	8.42E-10	0.3	3.86	12.95	1
cg27619163	ALOX12B	17	1.21E-09	0.39	6.64	16.94	1
cg14957718	SLC38A3	3	1.64E-09	0.3	3.73	12.57	1
cg20413471	ADAMTS19	5	1.94E-09	0.28	3.43	12.06	0.85
cg23661343	CLUL1	18	2.25E-09	0.49	11.76	23.9	0.9
cg24491576	POT1	7	3.67E-09	0.29	3.49	11.97	0.81
cg14345128	ONECUT2	18	3.89E-09	2.31	16.61	7.2	0.84
cg11377136	PKDREJ	22	4.87E-09	0.44	8.15	18.62	0.91
cg07675334	MAZ	16	9.52E-09	0.49	10.95	22.26	0.94
cg18051461	HIC1	17	1.02E-08	0.26	2.69	10.49	0.91
cg07115542	NKX2-1	14	1.13E-08	0.48	10.06	20.97	0.81
cg03384992	OSBPL1A	18	1.24E-08	0.26	2.76	10.54	0.83
cg15988350	PDE1C	7	1.26E-08	0.5	10.98	22.19	0.85
cg00839579	MEOX2	7	1.38E-08	0.47	9.13	19.61	0.81
cg26674752	CSGALNACT2	10	1.55E-08	0.39	5.77	14.86	0.93
cg17114847	AGBL2	11	2.25E-08	0.48	9.66	20.17	0.9
cg09874822	CACNA1H	16	2.69E-08	0.38	5.29	13.99	0.9
cg19870512	KCNA6	12	2.79E-08	0.43	7.14	16.6	0.85
cg23614791	ACSS3	12	3.02E-08	0.48	9.68	20.08	0.81
cg25277509	FGF9	13	3.41E-08	0.2	1.8	8.84	0.88
cg13196826	SLIT1	10	4.71E-08	0.25	2.36	9.57	0.85
cg00594560	ZFHX4	8	4.76E-08	0.4	5.89	14.65	0.88

**Fig 2:**
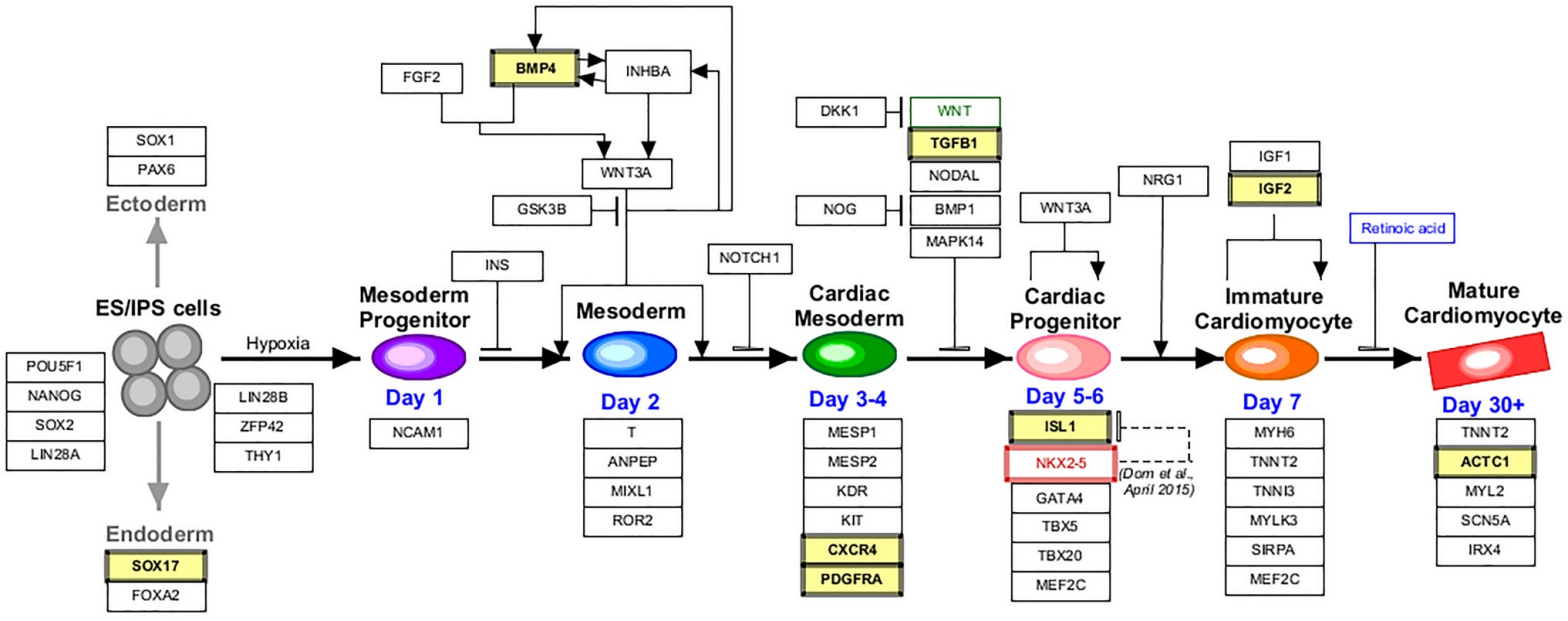
Schematic representation of the cardiac progenitor pathway. Previous studies identified several genetic components related to cardiac development and progression. Genes identified in the present study are shown are highlighted with yellow background.

Gene ontology and pathway analysis revealed an over-representation of pathways known to be involved in cardiac and ventricular chamber development (S1 Table). These genes included some known to be responsible for cardiac ventricle development (*HEY2*, FDR p = 0.002; *ISL1*, FDR p = 2.61E^-5^), cardiac septum formation (*ISL1*, FDR p = 2.61E^-5^), cardiac muscle development (*ACTC1*, FDR p = 7.15E^-12^) and others. In addition, Fig 2 displays pathways previously thought to be involved in cardiac development (Wikipathways). The associated legends indicate how to identify constituent genes found to be differentially methylated in the current study. Among the top differentially methylated genes were *BMP4*, *TGFB1*, *PDGFRA*, *TBX2*, *EGR1*, *SRF*, *CXCR4*, *TLX3*, *DPP6*, and *FGFR1*, known to be related to heart development or anomalies. In addition, the study also identified 8 microRNAs: miR-191, miR-548F1, miR-148A, miR-423, miR-92B, miR-611, miR-2110, and miR-548H4 (S2 Table). Significant differential methylation in the genes that coded for 52 Zinc finger proteins (S3 Table) were detected followed by 62 open reading frames (ORFs, S4 Table) and 28 LOCs (genes without a name and of unknown function, S5 Table) were detected. Additionally, differential gene methylation for 8 Small Nucleolar RNAs (SNOR, S6 Table) were identified and two non-coding RNAs (NCRNAs, S7 Table) were also identified. All CpG loci had individual AUC ROC ≥0.80 (at FDR p-value <0.001) for the detection of isolated VSD.

We also used conventional logistic regression to identify a parsimonious combination of genes for prediction of VSD. The combination of cg20739526 (*DPP6*) and cg19393008 (*KRT82*) markers had an AUC value of 0.997 with 95% CI (1.0, 1.0) (S2 Fig).

We did not perform gene expression studies correlated with CpG methylation levels. We did however perform GDAC FIREHOSE database analysis which showed that these methylation differences are correlated with altered gene expression in other tissues such as cancer cells (S8 and S9 Tables, S3 Fig). S9 Table shows the CpG targets, H3k27Ac modification, transcription factors and location of binding sites along with the statistical significance.

## Discussion

The placenta is now being recognized to play a “gate-keeper” role in cardiac development by way of multiple mechanisms [32]. There are hundreds of genes whose mutation produces both placental morphological and congenital heart defects [32]. The developing heart derives its supply of nutrients, including folate, from the placenta. Folate is the main source of the methyl group involved in the methylation of DNA. Folate is known to be protective of the placental-heart axis against environmental insults [9]. Several environmental insults are in turn known to play a critical role in the development of non-syndromic CHD. Folate deficiency has been linked to the development of CHD, primarily septal defects [33]. Other more striking examples of the role of the placenta in CHD development are commonly encountered in clinical obstetrics. There are two major arteries connecting the placenta to the fetal cardiovascular system, namely the umbilical arteries. Failure of development of one of the umbilical arteries (‘single umbilical artery’) is well-known to be associated with increased risk of several CHDs in the fetus [10]. Finally, blood flow, volume, and intra- cardiac pressures are known to play a decisive role in the development of CHD. In this context, the most dramatic example of the relationship of placental vascular abnormality and CHD is in twin-to-twin transfusion syndrome. This is a condition of monozygotic identical twins with monochorionic-diamniotic placentation. In monochorionic-diamniotic twins there is a single placenta in which there are extensive vascular connections between placental blood vessels, both arterial and venous, of each twin. In twin-to-twin transfusion syndrome, there is a gross transfer of blood volume through these connections favoring the “recipient” twin over the “donor” twin during monochorionic diamniotic twin gestation. As a consequence, there is a greater than 9-fold increase in CHD incidence in identical twins with monochorionic-diamniotic placentation compared to the general population, and the risk of CHD is more than two fold greater when monochorionic diamniotic twins develop twin-twin-transfusion syndrome compared to monochorionic diamniotic twins without twin to twin transfusion syndrome [34]. In twin-twin transfusion syndrome, treatment of placental vascular disorder with prenatal laser therapy is known to reverse some developing structural abnormalities of the heart. This confirms the important role of placental vascular development in the CHD pathogenesis. Further altered methylation of angiogenesis genes such as VEGF has been reported to regular placental angiogenesis [35]. Thus, providing a very credible link and potential mechanism by which the placenta can induce the development of CHD. For these reasons, we chose to evaluate placental epigenetics as a tool for understanding the pathogenesis of and prediction of non-symptomatic CHD, the dominant group of congenital heart defects.

We identified 1488 unique loci, one per gene, that were significantly differentially methylated in the placental DNA in cases of non-syndromic isolated VSD compared to controls. The methylation status of the CpG sites had high predictive accuracy for VSD detection with AUC ≥0.80. Indeed 80 of the cytosine loci in 80 genes had AUC = 1.0 at the FDR p-value <0.005. In addition, significant differential methylation was noted in genes coding for many ORFs, NCRAs and LOCs. These also demonstrated good to excellent diagnostic accuracy for VSD detection. We also identified methylation changes in 8 miRNAs that modulate the activity of 160 genes identified.

### Cardiac progenitors and their role in heart development

Pathway analysis identified multiple genes known to be involved in cardiac development overall, and ventricular development and cardiac septum formation. These associations give biological plausibility to our findings (S1 Table). Recent work indicates that a common multipotent stem cell differentiates into cardiomyocyte, smooth muscle, and endothelial lineages, the major differentiated cell types of the heart [36–38]. These precursor cells are characterized by expression of the transcription factors Isl1 and Nkx2-5. Lineage tracing studies indicate that Isl1 and Nkx2-5 expression contributes to differentiation of the cardiomyocytes and a subset of the smooth muscle and endothelial cells of the heart [38]. Both genes were differentially methylated in CHD cases compared to controls in our study. We will now briefly discuss some of the genes and pathways found to be epigenetically dysregulated in our study and possible biological significance in CHD and VSD development.

### TGF-β signaling in cardiomyocyte differentiation

The TGF-β superfamily members critically regulate many different processes within the cardiovascular system (CVS), including heart development and angiogenesis. The predominant TGF-β superfamily ligands expressed in the CVS are *TGF-β1*, *TGF-β2*, *TGF-β3*, *BMP-2*, *BMP-4*, *BMP-6*, and BMP-7 [39]. The importance of the *TGF-β* superfamily in cardiovascular development is demonstrated by the significant phenotypic changes in knock-out mice models. Disturbances in TGF-β signaling in mice causes embryonic lethality and the development of VSDs, myocardial thinning, and double outlet right ventricle (DORV), a cardiac anomaly of which VSD is a feature [40]. Moreover, BMP-dependent activation of transcription factors including the cardiac zinc finger transcription factor *GATA4*, homeobox protein *NKX2*.*5*, and myocyte enhancer factor 2C (MEF2C) augment differentiation mediated by the SMAD signaling pathways that regulate cardiomyocyte proliferation [41].

### MicroRNAs and cardiac diseases

MicroRNAs (miRNAs) are endogenous, small, evolutionary conserved, non-coding RNAs molecules about 22 nucleotides in length that function in the regulation of gene expression [42]. They bind to and inactivate mRNA thus inhibiting protein production. miRNAs are commonly found in clusters through many different regions of the genome, most frequently within intergenic regions and the introns of protein-coding genes [43]. Altered miRNAs expression is known to occur in various cardiovascular disorders such as hypertension, congestive heart failure, CHD, coronary artery disease and stroke. In the present study, significant changes in the methylation of several miRNA genes: miR-191, miR-548f1, miR-148a, miR-423, miR-92b, miR-611, miR-2110, and miR-548h4 were observed in the placenta of VSD subjects as compared to controls. Our findings are supported by previous publications indicating these microRNAs are associated with cardiac disorders including VSD and heart failure [44–46].

Chen et al [47] suggested that miR-92b is involved in visceral and cardiac muscle differentiation and regeneration. Additionally, significant changes in miR-92b expression levels have been reported in neointima formation in a rat model of vascular injury [48], in individuals with heart failure [49], and during cardiac rehabilitation following surgical coronary revascularization [50], suggesting its involvement in these processes. In the present study, a significant variation in placental gene methylation was observed in VSD cases compared to controls.

### Fetal / Embryonic cardiac muscle formation

The *ACTC1* (Actin, Alpha, Cardiac Muscle 1) gene on chromosome 15q14 (MIM 102540) is the only actin expressed in embryonic heart muscle [51]. It has been suggested that the lack of *ACTC1* may induce apoptosis, leading to disrupted cardiac differentiation. Apoptosis plays a crucial role in embryological development and excessive absorption/apoptosis of the primary septum is thought to be a cause of atrioventricular septal defects [52].

Jak / stat signaling in cardiac remodeling and proliferation:

One study found that Jak1/Stat3 downstream mediators are induced upon cardiac injury. Inhibition of Stat3 signaling in cardiomyocytes using a dominant-negative transgene led to fibrotic scarring and reduced cardiomyocyte proliferation [53].

Other significant genes involved in cardiac development:

Recent evidence has pointed to *IGF2* involvement in cardiac development. *IGF2* is expressed by epicardial cells during mid-gestation murine heart development, and IGF2-deficient embryos show reduced cardiomyocyte proliferation in the ventricular wall. Importantly, the cardiomyocyte-specific deletion of the genes encoding the *IGF2* receptors IGF1R and Insr replicated this effect [54].

The inactivation of *Hey2*, a significant Notch signaling molecule, in mice resulted in a spectrum of cardiac malformations that resembled those associated with mutations of human *JAG1* [55], associated with Tetralogy of Fallot (TOF), VSD, and tricuspid atresia. Sakata et al [8] reported that targeted disruption of the *CHF1/Hey2* locus in mice resulted in VSDs.

Bone Morphogenetic Protein 4 (*BMP4*) on 14q22.2 region is a member of the TGF-β signaling family. The effect of BMPs are exerted through receptor-mediated activation of the transcription factor Smad by binding to heterotetrameric receptor complexes [56]. BMPs can induce formation of bone and cartilage, and play a major role in embryogenesis [57]. *BMP4* plays a key role in cardiac development, with expression in ventral splanchnic arteries, branchial-arch mesoderm and outflow tract myocardium underlying the cushion-forming regions of the heart [58,59]. *BMP4* alterations are known to be associated with defects in cardiac septation such as atrial septal defect (ASD), VSD, and atrioventricular septal defects (AVSD). The inactivation of BMP4 results in neonatal lethality [60,61].

Isl Lim Homeobox 1 (ISL1) on chromosome 5q11.1 is a transcription factor important in multipotent cardiovascular cell lineages [62]. The lineages of Isl1-expressing cells include neural crest, endocardium, endothelium, myocardium and smooth muscle cells [63]. Genetic mutations of ISL1 have been found to be associated with CHD and VSD [62,64].

Platelet-Derived Growth Factor Receptor, Alpha (*PDGFR-A*) proteins are receptor tyrosine kinases (RTKs) coded on chromosome 4q12 and are important in PDGF signaling. They specifically localize in the primary cilium of the heart where the downstream effectors PI3K–AKT and MEK1/2–ERK1/2 are active and regulate processes like cell cycle control and cell migration in fibroblasts [65,66]. Cilia play a major role in signaling processes which contribute to the left-right organ asymmetry, differentiation, morphogenesis and the maturation of the heart [66].

During heart development, *T-BOX 2* (*TBX2*) on chromosome 17q23.2 is expressed in the outflow and inflow tracts, inner curvature and atrioventricular canal [67]. Sequence variants at the promoter sequences of *TBX2* have been identified in VSD patients [6].

Early Growth Response 1 (*EGR1*) factor gene on chromosome 5q31.2 controls proliferation, differentiation, and apoptosis of cells, and is also involved in hypoxic and ischemic cardiovascular injuries [68]. *EGR1* is a transcription factor for *NAB1* and is involved in the regulation of physiological and pathological hypertrophy of the heart. *Egr1* may not be required for normal development of muscle mass, but it acquires functional importance during the response to cardiac stress resulting from mechanical overload and hypertrophic signaling [69].

*DPP6* is an essential component of the native cardiac channel complex (Kv4.3, *KCND3*) which regulates cardiac conduction [70]. *DPP6* may serve as an additional beta-subunit responsible for the transient outward current generated during repolarization of the nodal cells in the human heart. Kv4.3, an outward flux potassium ion channel, is expressed extensively in ventricular myocytes.

A significant amount of inactive CaMKII forms a molecular complex with Kv4.3 potassium ion channel proteins in cardiomyocytes as a CaMKII reservoir [71]. CaMKII is also a positive regulator of T-Type calcium ion channels, one of which is *CACNA1H [72]*. This channel is responsible for transient calcium ion influx current (I_Ca,T_) into myocardial cells following depolarization [72]. In ventricular myocardium, the T-type Ca^2+^ current channel *CACNA1H*, which is temporarily observed during fetal and neonatal periods, has been shown to reappear in failing/remodeling hearts. These findings suggest that *CACNA1H* is related to cell growth, proliferation, and development [73].

LOC gene(s) are predicted genes whose functions are uncharacterized, and that are currently unnamed. They are assigned to a generic gene and protein category “uncharacterized LOC” plus the GeneID [45]. In the present analysis, we have identified 28 LOC genes on 14 chromosomal regions (S5 Table). Each of these 26 CpG different targets have a ROC AUC ≥0.81 and the FDR p-value <0.005 for VSD prediction. Among these 28 candidates, LOC374443 [74], LOC255512 [75], LOC348926 [76], LOC96610 [77], LOC25845 [78] and LOC220930 [79] expression are associated with different heart ailments. LOC150381 was previously identified under rare Copy Number Variation (CNV) deletion associated with aortic valve stenosis [80].

As previously noted, though DNA methylation patterns are largely tissue specific recent data suggests correlation between disease-induced methylation changes across tissues [81]. For example, a high degree of correlation in CpG methylation was demonstrated between blood leucocyte and tissue DNA from the lip in cleft lip and palate patients [17]. This phenomenon of cross tissue correlation of methylation markers is the most likely explanation for the observed epigenetic modification in cardiac development genes in the placental of non-syndromic VSD cases.

Our study does have some limitations, one of which is the small sample size. We are now unable to evaluate the role of potential confounders (e.g. diet, obesity, and ethnicity) on methylation patterns. Our study, however, is a proof-of-concept study whose purpose is to establish the plausibility of a concept. The next step will be to explore this phenomenon in a larger study population.

While it is widely recognized that DNA methylation correlates with gene expression, we did not evaluate this correlation in our study. This is clearly an area for future study. While we did not perform gene expression studies, GDAC FIREHOSE database analysis suggested that methylation levels in many of our loci correlate with gene expression at least in other tissues (S8 and S9 Tables, S3 Fig).

## Conclusions

To the author’s knowledge, this is the first study reporting the use of placental molecular markers for CHD detection and for elucidating the mechanism of CHD. We report significant epigenetic dysregulation in the placental DNA of VSD cases. Multiple genes and gene pathways including some known or suspected to be involved in heart and ventricular development were affected. These findings could have scientific and clinical significance in furthering understanding the pathogenesis of CHD and in the development of accurate biomarkers for CHD using surrogate tissues such as placenta. Placental tissue is clinically accessible in both the first and early second trimester when diagnosis of CHD is of optimal value. Further, placental tissue obtained at birth could also be used for the prediction of newborn CHD. Newborn screening for critical CHD is now a standard of care.

## Supporting information

S1 Fig (Online only) Receiver operating characteristic (ROC) curve analysis of methylation profiles for four specific markers associated with VSD. The study identified 1328 CpG sites in 1328 genes with significantly differentially-methylated genes that have an area under the ROC curve ≥0.80. At each locus, the False Detection Rate p-value for the methylation difference between VSD subjects and controls was highly significantly different. Due to figure resolution concerns, we have included only four markers (chr 21; cg21161649) (chr 17; cg04245057) (chr 4; cg07809452) (chr 12; cg22129822). AUC: Area Under the Receiver Operating Characteristics Curve; 95% CI: 95% Confidence Interval. Lower and upper confidence intervals are given in parentheses.
(PDF)

S2 Fig (Online only). Logistic regression analysis of VSD cases and controls. (PNG)

S3 Fig (Online only) Open chromatin and transcription factor occupancy in functional CpG sites. Pie charts depicting H3K27Ac histone mark layering, location of CpG sites and transcription factor binding sites.
(PDF)

S1 Table (Online only) Gene Ontology (GO) terms enriched among the genes in the network displayed by GeneMANIA. (PDF)

S2 Table (Online only) Differentially methylated CpG sites with Target ID, Gene ID, chromosome location, FDR p-value, and % methylation change in VSD cases and controls for each target methylated for MicroRNA. (PDF)

S3 Table (Online only) Differentially methylated CpG sites with Target ID, Gene ID, chromosome location, FDR p-value, and % methylation change in VSD cases and controls for each target methylated for Zinc finger genes. (PDF)

S4 Table (Online only) Differentially methylated CpG sites with Target ID, Gene ID, chromosome location, FDR p-value, and % methylation change in VSD cases and controls for each target methylated for open reading frame genes (ORFs). (PDF)

S5 Table (Online only) Differentially methylated CpG sites with Target ID, Gene ID, chromosome location, FDR p-value, and % methylation change in VSD cases and controls for each target methylated for LOC (uncharacterized) genes. (PDF)

S6 Table (Online only) Differentially methylated CpG sites with Target ID, Gene ID, chromosome location, FDR p-value, and % methylation change in VSD cases and controls for each target methylated for small non-coding RNA (SNOR) genes. (PDF)

S7 Table (Online only) Differentially methylated CpG sites with Target ID, Gene ID, chromosome location, FDR p-value, and % methylation change in VSD cases and controls for each target methylated for non-coding RNA genes (NCRNAs). (PDF)

S8 Table (Online only) Correlation of methylation mean with expression mean in various human tissues from GDAC data. 180 differentially methylated CpG targets were correlated with expression (RNA-seq) data. A bar chart was generated for each CpG target showing the proportion of methylation and mean of expression of the gene in which the CpG target resided.
(PDF)

S9 Table (Online only) Open chromatin conformation and transcription factor binding in differentially methylated CpG sites indicated their role in transcription initiation. ENCODE data showing the H3K27Ac layering on each CpG site presenting an open chromatin conformation. These CpG targets were also occupied with various transcription initiation factors, mostly PolR2A. The position of each CpG site was also noted in respect to the gene in which it resided. Some of the differentially methylated CpG sites that were resided in intronic or 1^st^ exonic regions, signifying their essential function in modulating transcription.
(PDF)
