# Supplementary figures and images for: Placental epigenetics for evaluation of fetal congenital heart defects: Ventricular Septal Defect (VSD)
Source: PLoS One. 2019 Mar 21;14(3):e0200229. doi: 10.1371/journal.pone.0200229 (PMC6428297; doi:10.1371/journal.pone.0200229)

**Chr 21; cg21161649 (*AGPAT3*)**

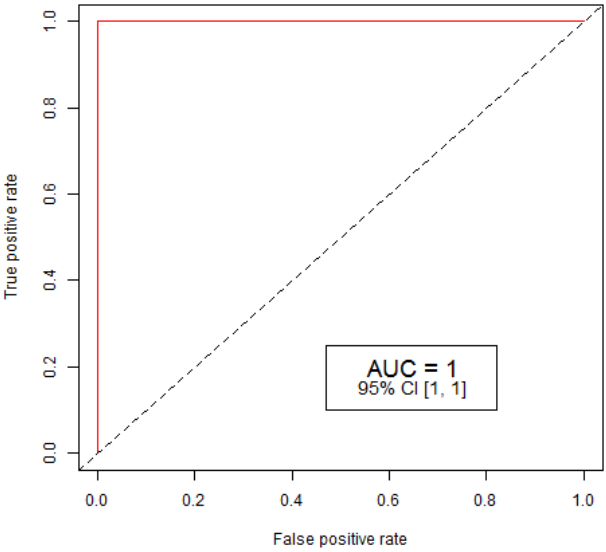

**Chr 17; cg04245057 (*TBX2*)**

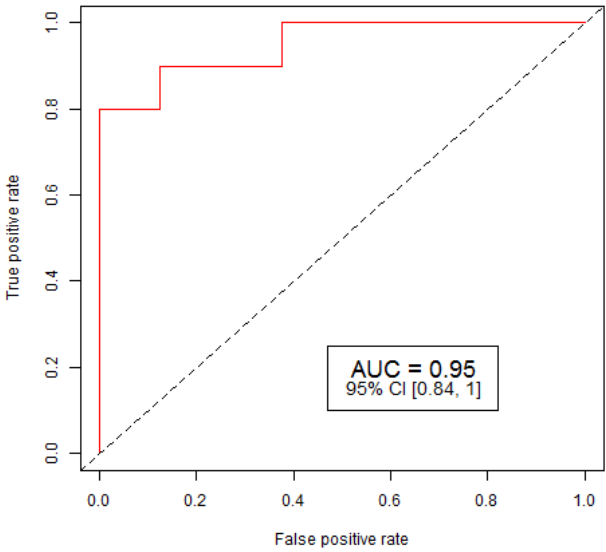

**Chr 4; cg07809452 (*FGFRL1*)**

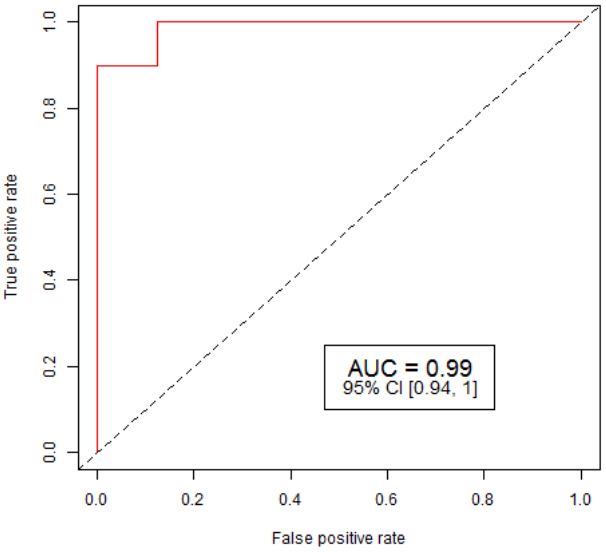

**Chr 12; cg22129822 (*MARS*)**

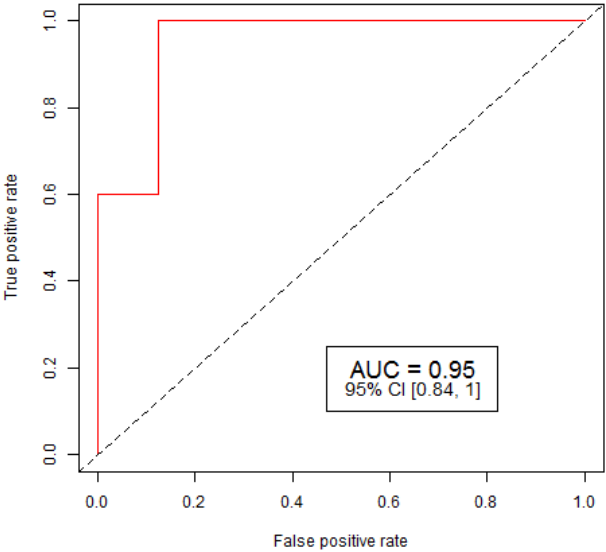

Supplement: S1 Fig — The study identified 1328 CpG sites in 1328 genes with significantly differentially-methylated genes that have an area under the ROC curve ≥0.80. At each locus, the False Detection Rate p-value for the methylation difference between VSD subjects and controls was highly significantly different. Due to figure resolution concerns, we have included only four markers (chr 21; cg21161649) (chr 17; cg04245057) (chr 4; cg07809452) (chr 12; cg22129822). AUC: Area Under the Receiver Operating Characteristics Curve; 95% CI: 95% Confidence Interval. Lower and upper confidence intervals are given in parentheses. (PDF) [file pone.0200229.s001.pdf]

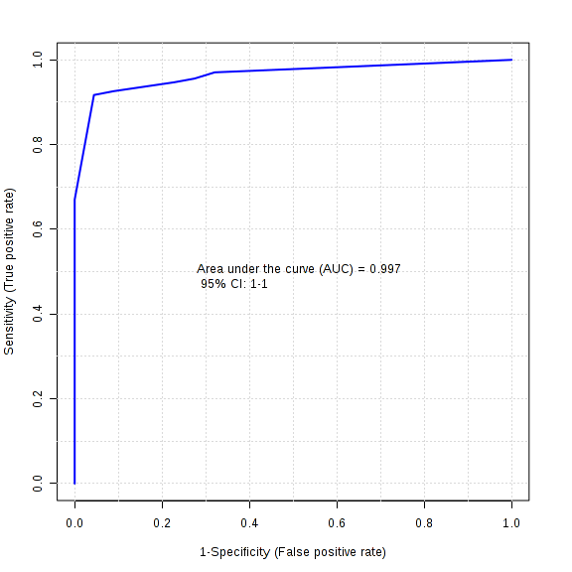

Supplement: S2 Fig — (PNG) [file pone.0200229.s002.png]

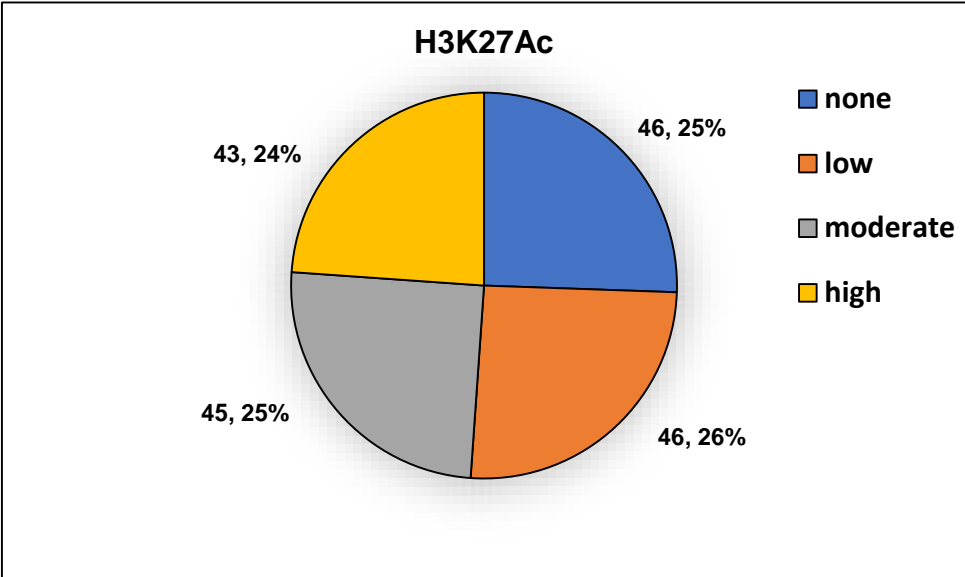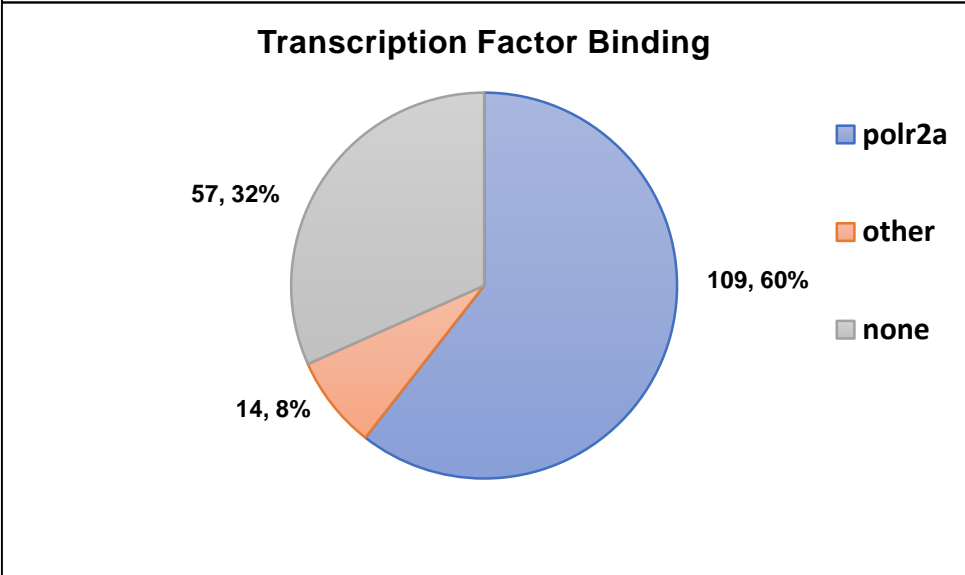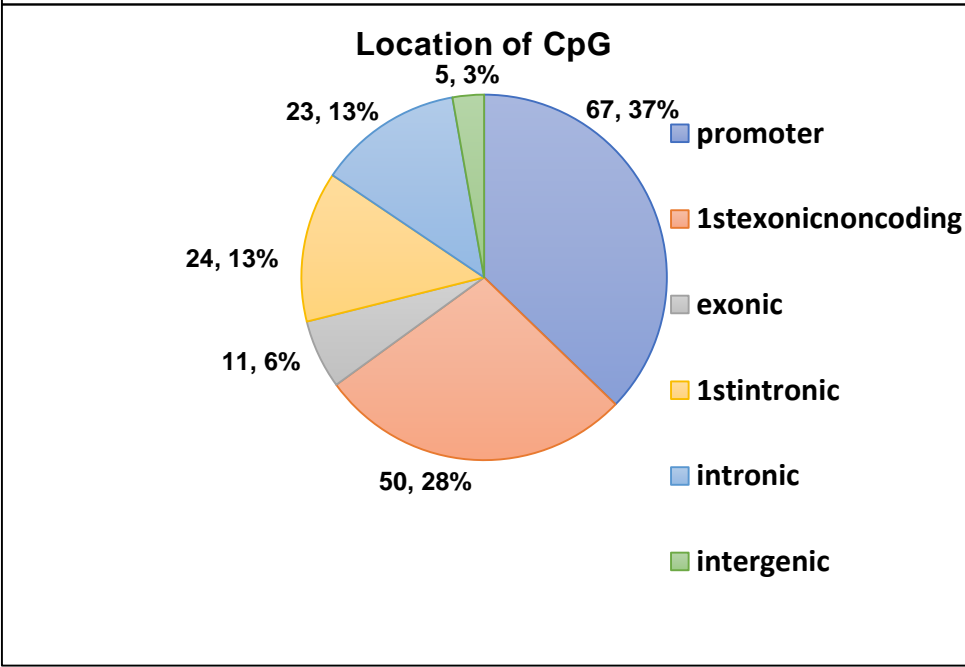

Supplement: S3 Fig — Pie charts depicting H3K27Ac histone mark layering, location of CpG sites and transcription factor binding sites. (PDF) [file pone.0200229.s003.pdf]
