# Supplementary material for: Placental epigenetics for evaluation of fetal congenital heart defects: Ventricular Septal Defect (VSD)
Source: PLoS One. 2019 Mar 21;14(3):e0200229. doi: 10.1371/journal.pone.0200229 (PMC6428297; doi:10.1371/journal.pone.0200229)
Supplement: S1 Table — (PDF) [file pone.0200229.s004.pdf]

Pathways	*Genes	Significance
Cardiac ventricle development	HEY2, ISL1	1.66E-18
Vasculature development	HEY2, SRF	1.59E-12
Sequence-specific DNA binding	TBX2, HEY2, HEYL, ISL1	1.20E-10
Heart looping	SRF	1.74E-10
Cardiac muscle cell differentiation	ACTC1, HEY2	1.21E-08
Cellular response to endogenous stimulus	ISL1	1.26E-08
Angiogenesis	SRF	1.14E-04
Embryonic heart tube development	SRF	1.48E-11
Heart contraction	ACTC1	1.78E-03
Cardiac septum development	ISL1	2.53E-16
Heart morphogenesis	SRF, HEY2, ISL1, HEYL	2.70E-30
Cardiac muscle tissue development	ACTC1, ISL1	2.82E-23
Pattern specification process	ISL1	3.40E-20
Notch signaling pathway	HEY2, HEYL	4.33E-08
Blood vessel morphogenesis	SRF	4.34E-09
Blood vessel development	HEY2, SRF	6.06E-12
Ras protein signal transduction	RAF1	8.04E-07
Cardiac chamber development	ISL1	8.17E-23
Cardiac chamber morphogenesis	HEY2, ISL1	8.79E-22

*We have identified these constituent genes to be differentially methylated in the present study
