# Supplementary material for: Placental epigenetics for evaluation of fetal congenital heart defects: Ventricular Septal Defect (VSD)
Source: PLoS One. 2019 Mar 21;14(3):e0200229. doi: 10.1371/journal.pone.0200229 (PMC6428297; doi:10.1371/journal.pone.0200229)
Supplement: S2 Table — (PDF) [file pone.0200229.s005.pdf]

Target ID	Gene ID	CHR	LOG10p	FDR p-Val	Fold change	% Methylation Cases	% Methylation Control	AUC
cg19526908	MIR191	3	-4.28	5.25E-05	0.26	1.66	6.50	1.0
cg22865402	MIR548F1	1	-2.69	0.0020	0.20	0.83	4.20	0.93
cg25771013	MIR148A	7	-9.28	5.19E-10	0.46	10.48	22.67	0.91
cg24102938	MIR423	17	-3.92	0.0001	0.33	2.42	7.24	0.88
cg12678006	MIR92B	1	-2.10	0.0079	0.34	1.54	4.58	0.88
cg26061001	MIR611	11	-3.01	0.0010	0.43	3.31	7.62	0.84
cg15372479	MIR2110	10	-2.23	0.0059	0.42	2.44	5.80	0.84
cg07057074	MIR548H4	15	-2.98	0.0011	0.29	1.53	5.32	0.83
