# Supplementary material for: Placental epigenetics for evaluation of fetal congenital heart defects: Ventricular Septal Defect (VSD)
Source: PLoS One. 2019 Mar 21;14(3):e0200229. doi: 10.1371/journal.pone.0200229 (PMC6428297; doi:10.1371/journal.pone.0200229)
Supplement: S3 Table — (PDF) [file pone.0200229.s006.pdf]

Target ID	Gene ID	CHR	FDR p-Val	Fold change	% Methylation Cases	% Methylation Control	AUC
cg10001458	ZNF33B	10	1.78E-12	0.32	5.56	17.28	0.85
cg25151919	ZNF502	3	1.63E-09	0.39	6.45	16.57	0.81
cg14376988	ZNF367	9	9.22E-06	0.49	7.00	14.25	0.86
cg20778451	ZNF454	5	1.3E-05	0.36	3.27	9.14	0.93
cg23267217	ZNF593	1	1.55E-05	0.27	1.97	7.30	0.89
cg24387962	ZNF770	15	3.52E-05	0.30	2.25	7.42	0.88
cg26263766	ZNF544	19	4.52E-05	0.22	1.34	6.11	0.85
cg22079684	ZNF416	19	4.56E-05	0.36	3.05	8.42	0.91
cg27545919	ZNF461	19	5.59E-05	0.23	1.45	6.20	0.93
cg08867893	ZNF365	10	5.95E-05	0.45	4.77	10.60	0.84
cg11684022	ZNF496	1	6.61E-05	0.45	4.82	10.61	0.90
cg04154138	ZNF677	19	6.98E-05	0.42	3.95	9.45	0.83
cg02295078	ZNF287	17	7.99E-05	0.45	4.63	10.30	0.89
cg19342764	ZNF761	19	9.77E-05	0.18	0.97	5.38	0.91
cg16442574	ZNF341	20	0.0001	0.26	1.65	6.26	0.84
cg14556070	ZNF256	19	0.0002	0.43	3.94	9.11	0.81
cg18154014	ZNF793	19	0.0002	0.32	2.18	6.80	0.85
cg13009365	ZNF70	22	0.0002	0.42	3.69	8.75	0.86
cg13536757	ZNF804A	2	0.0002	0.24	1.35	5.64	0.85
cg22076081	ZNF133	20	0.0002	0.46	4.29	9.42	1.0
cg24357026	ZNF565;ZNF146	19	0.0003	0.34	2.39	6.93	0.90
cg22657780	ZNF385B	2	0.0003	0.37	2.70	7.25	0.91
cg03004280	ZNF75A	16	0.0004	0.49	5.01	10.15	0.81
cg03235119	ZNF346	5	0.0004	0.44	3.74	8.48	0.84
cg17289202	ZNF532	18	0.0005	0.11	0.47	4.23	0.83
cg20451226	ZNF155	19	0.0005	0.35	2.27	6.55	0.90
cg01421963	ZNF441	19	0.0005	0.44	3.62	8.26	0.90
cg19709878	ZNF652	17	0.0005	0.42	3.32	7.87	0.81
cg07515940	ZNF354C	5	0.0005	0.47	4.32	9.13	0.91
cg00875960	ZNF625	19	0.0006	0.44	3.52	8.09	0.85
cg11281912	ZNF585B	19	0.0006	0.35	2.24	6.42	0.86
cg18542992	ZNF703	8	0.0009	0.49	4.47	9.13	0.84
cg00655071	ZNF280D	15	0.0009	0.37	2.46	6.57	0.83
cg16242708	ZNF780A	19	0.0010	0.50	4.64	9.29	0.89
cg22852149	ZNF566	19	0.0010	0.30	1.68	5.53	0.83
cg24150232	ZNF264	19	0.0010	0.49	4.35	8.92	0.90
cg25335229	ZNF616	19	0.0010	0.49	4.46	9.04	0.94
cg21961831	ZNF599	19	0.0010	0.34	2.00	5.93	1.0
cg23255774	ZNF460	19	0.0012	0.49	4.28	8.75	0.93

cg20744304	ZNF433	19	0.0012	0.37	2.26	6.20	0.81
cg20640573	ZNF101	19	0.0018	0.45	3.27	7.31	0.81
cg17478827	ZNF524	19	0.0022	0.48	3.75	7.84	0.89
cg11037064	ZNF318	6	0.0030	0.43	2.84	6.57	0.85
cg02517932	ZNF282	7	0.0032	0.41	2.56	6.21	0.89
cg24179850	ZNF581	19	0.0038	0.49	3.74	7.58	0.85
cg15427393	ZNF575	19	0.0042	0.38	2.05	5.46	0.84
cg11737710	ZNF570	19	0.0046	0.50	3.71	7.47	0.90
cg17279338	ZNF69	19	0.0047	0.47	3.23	6.86	0.89
cg24911123	ZNF540;ZNF571	19	0.0061	0.35	1.73	4.91	0.88
cg15248304	ZNF485	10	0.0073	0.50	3.43	6.92	0.88
cg01268683	ZNF83	19	0.0092	0.49	3.21	6.54	0.81
cg03579738	ZNF57	19	0.0098	0.31	1.29	4.18	0.83
