# Supplementary material for: Placental epigenetics for evaluation of fetal congenital heart defects: Ventricular Septal Defect (VSD)
Source: PLoS One. 2019 Mar 21;14(3):e0200229. doi: 10.1371/journal.pone.0200229 (PMC6428297; doi:10.1371/journal.pone.0200229)
Supplement: S4 Table — (PDF) [file pone.0200229.s007.pdf]

Target ID	Gene ID	CHR	FDR p-Val	Fold chance	% Methylation Cases	% Methylation Control	AUC
cg19679633	C5orf38	5	0.001295	0.49	4.31	8.76	1.0
cg19950069	C17orf101	17	9.11E-05	0.44	4.43	9.99	1.0
cg27594176	C17orf91	17	6.97E-06	0.25	1.81	7.33	1.0
cg15790941	C4orf34	4	0.000781	0.36	2.34	6.46	1.0
cg00310855	C11orf2	11	9.62E-07	0.43	5.97	13.72	0.94
cg13139203	C11orf84	11	7.76E-05	0.37	3.03	8.19	0.94
cg14452706	C14orf73	14	0.000238	0.35	2.45	7.03	0.94
cg17473382	C17orf82	17	2.17E-06	0.41	4.90	11.97	0.94
cg00125159	C1orf35	1	4E-06	0.42	4.92	11.79	0.9
cg19244640	C1orf212	1	6.02E-06	0.31	2.63	8.50	0.93
cg17973565	C1orf77	1	1.87E-05	0.35	3.06	8.72	0.93
cg13771733	C9orf114	9	0.000206	0.25	1.41	5.72	0.93
cg08904082	C5orf43	5	2.26E-06	0.13	0.84	6.30	0.91
cg11698382	C6orf136	6	0.002808	0.39	2.37	6.01	0.90
cg07692169	C16orf13	16	0.000659	0.46	4.03	8.69	0.89
cg09654157	C20orf96	20	2.18E-05	0.17	1.01	5.89	0.89
cg14558431	C9orf170	9	0.000109	0.18	0.96	5.34	0.89
cg25424237	C12orf65	12	0.000745	0.44	3.57	8.06	0.88
cg08187083	C17orf79	17	0.001559	0.48	3.88	8.15	0.88
cg11352290	C2orf18	2	0.000486	0.31	1.91	6.09	0.88
cg14174901	C2orf76	2	3.89E-05	0.40	3.73	9.37	0.88
cg03386905	C5orf45	5	0.005102	0.34	1.70	4.95	0.88
cg20054027	C11orf80	11	0.000256	0.49	5.27	10.66	0.88
cg09610071	C16orf93	16	0.007012	0.47	3.07	6.49	0.88
cg17660384	C11orf70	11	0.000386	0.31	1.91	6.17	0.86
cg14698297	C11orf80	11	0.001026	0.31	1.71	5.56	0.86
cg15731056	C12orf56	12	0.006214	0.39	2.05	5.30	0.86
cg05344495	C19orf77	19	3.7E-07	0.49	8.70	17.79	0.86
cg09091443	C5orf24	5	2.57E-10	0.11	0.92	8.73	0.86
cg07660862	C6orf136	6	0.000328	0.49	4.95	10.15	0.86
cg12733656	C7orf70	7	0.003552	0.43	2.76	6.41	0.86
cg26277709	C9orf3	9	0.005313	0.44	2.74	6.21	0.86
cg16529530	C1orf91	1	0.003303	0.49	3.68	7.58	0.86
cg23206103	C10orf118	10	0.000792	0.20	0.92	4.65	0.85
cg07469949	C19orf62	19	0.000756	0.25	1.31	5.15	0.84
cg23318713	C10orf55	10	0.001331	0.49	4.29	8.73	0.84
cg14868663	C14orf133	14	0.002785	0.36	2.00	5.56	0.83
cg04749771	C14orf149	14	0.000419	0.42	3.37	8.03	0.83
cg14981312	C1orf159	1	0.009564	0.50	3.29	6.62	0.83
cg11520162	C1orf93	1	0.003787	0.43	2.79	6.41	0.83

cg05722611	C22orf41	22	6.44E-06	0.31	2.67	8.54	0.83
cg02941781	C2orf88	2	0.002386	0.44	3.02	6.89	0.83
cg07908870	C3orf59	3	0.000576	0.38	2.66	6.99	0.83
cg09039647	C5orf15	5	2.02E-06	0.31	2.88	9.20	0.83
cg27368511	C5orf54	5	0.004176	0.47	3.30	7.00	0.83
cg14525935	C7orf50	7	0.003252	0.47	3.35	7.17	0.83
cg06443863	C8orf33	8	0.002027	0.47	3.66	7.76	0.83
cg13529074	C8orf41	8	0.000115	0.40	3.44	8.60	0.83
cg14274895	C9orf130	9	8.49E-11	0.14	1.37	9.70	0.83
cg23063986	C4orf29	4	0.001488	0.41	2.76	6.76	0.83
cg07226793	C16orf93	16	0.000614	0.37	2.54	6.81	0.83
cg15155738	C12orf43	12	0.00029	0.29	1.71	6.01	0.81
cg25410318	C15orf39	15	0.003492	0.13	0.46	3.54	0.81
cg25194055	C17orf44	17	0.00343	0.49	3.83	7.75	0.81
cg23637705	C17orf61	17	0.000794	0.25	1.27	5.09	0.81
cg03339469	C19orf61	19	0.000259	0.19	0.93	5.02	0.81
cg00461735	C1orf31	1	0.000242	0.36	2.57	7.19	0.81
cg08614659	C21orf33	21	0.000657	0.37	2.47	6.71	0.81
cg00746515	C4orf27	4	0.009047	0.49	3.25	6.60	0.81
cg18136932	C5orf49	5	8.94E-05	0.28	1.89	6.63	0.81
cg16324746	C6orf72	6	0.002517	0.47	3.57	7.56	0.81
cg04619859	C7orf49	7	1.68E-05	0.20	1.30	6.36	0.81
