# Supplementary material for: Placental epigenetics for evaluation of fetal congenital heart defects: Ventricular Septal Defect (VSD)
Source: PLoS One. 2019 Mar 21;14(3):e0200229. doi: 10.1371/journal.pone.0200229 (PMC6428297; doi:10.1371/journal.pone.0200229)
Supplement: S5 Table — (PDF) [file pone.0200229.s008.pdf]

Target ID	Gene ID	CHR	FDR p-Val	Fold change	% Methylation Cases	% Methylation Control	AUC
cg02067211	LOC100133091	7	6.11676E-10	0.15	1.34	9.19	0.81
cg00785112	LOC150381	22	2.09568E-07	0.47	8.10	17.20	0.88
cg18433402	LOC389332	5	9.91034E-07	0.23	1.85	7.97	0.85
cg07089783	LOC220930	10	1.54937E-05	0.48	6.47	13.35	0.88
cg08981282	LOC652276	16	2.225E-05	0.34	2.87	8.41	0.90
cg17284070	LOC25845	5	3.49184E-05	0.27	1.82	6.85	0.91
cg00568641	LOC100268168	5	4.91023E-05	0.39	3.58	9.09	0.86
cg19342764	LOC147804	19	9.77181E-05	0.18	0.97	5.38	0.91
cg26658439	LOC81691	16	9.92358E-05	0.32	2.34	7.20	0.90
cg26182254	LOC145783	15	0.0001002	0.46	4.69	10.29	0.90
cg06123783	LOC441046	4	0.0001550	0.27	1.70	6.20	0.83
cg16110940	LOC400891	22	0.0003069	0.46	4.29	9.33	0.81
cg18347226	LOC285375	3	0.0003108	0.26	1.49	5.69	0.84
cg14231987	LOC80054	19	0.0003329	0.47	4.42	9.46	0.86
cg14328782	LOC92659	17	0.0003957	0.42	3.43	8.13	0.88
cg26340994	LOC729375	3	0.0005205	0.30	1.73	5.83	0.83
cg26116741	LOC96610	22	0.0006062	0.49	4.60	9.45	0.84
cg22905859	LOC728855	1	0.0006092	0.29	1.64	5.66	0.89
cg21349645	LOC642846	12	0.0009847	0.42	3.05	7.29	0.85
cg17644311	LOC144486	12	0.0015583	0.30	1.53	5.18	0.86
cg14379854	LOC91149	2	0.0017229	0.48	3.86	8.08	0.89
cg13590336	LOC145783	15	0.0019352	0.36	2.11	5.84	0.83
cg21322654	LOC286002	7	0.0021593	0.50	4.19	8.39	0.89
cg21485132	LOC348926	4	0.0021662	0.26	1.19	4.63	0.85
cg07441654	LOC255512	11	0.0024897	0.26	1.20	4.59	0.81
cg02963327	LOC374443	12	0.0024983	0.48	3.77	7.81	0.81
cg01992603	LOC348926	4	0.0027923	0.30	1.46	4.88	0.81
cg10884867	LOC100189589	2	0.0051559	0.46	2.95	6.48	0.85
