# Supplementary material for: Placental epigenetics for evaluation of fetal congenital heart defects: Ventricular Septal Defect (VSD)
Source: PLoS One. 2019 Mar 21;14(3):e0200229. doi: 10.1371/journal.pone.0200229 (PMC6428297; doi:10.1371/journal.pone.0200229)
Supplement: S6 Table — (PDF) [file pone.0200229.s009.pdf]

Target ID	Gene ID	CHR	FDR p-Val	Fold change	% Methylation Cases	% Methylation Control	AUC
cg26214747	SNORA13	5	6.14961E-05	0.49	6.02	12.21	0.91
cg11891983	SNORD58A	18	0.0013	0.36	2.23	6.13	0.91
cg09585247	SNORD101	6	7.67367E-05	0.26	1.63	6.34	0.90
cg13595191	SNORD43	22	0.0014	0.29	1.47	5.16	0.90
cg00210098	SNORA7A	3	0.0030	0.38	2.14	5.70	0.86
cg01035068	SNORD68	16	5.27596E-05	0.39	3.45	8.89	0.85
cg08688063	SNORA80B	2	0.0002	0.19	0.96	5.07	0.84
cg21595175	SNORD95	5	0.0004	0.31	1.86	6.06	0.84
