# Supplementary material for: Placental epigenetics for evaluation of fetal congenital heart defects: Ventricular Septal Defect (VSD)
Source: PLoS One. 2019 Mar 21;14(3):e0200229. doi: 10.1371/journal.pone.0200229 (PMC6428297; doi:10.1371/journal.pone.0200229)
Supplement: S7 Table — (PDF) [file pone.0200229.s010.pdf]

Target ID	Gene ID	CHR	FDR p-Val	Fold change	% Methylation Cases	% Methylation Control	AUC
cg01551879	NCRNA00115	1	7.46725E-05	0.33	2.44	7.43	0.85
cg26214747	NCRNA00219	5	6.14961E-05	0.49	6.02	12.21	0.91
