# Supplementary material for: Placental epigenetics for evaluation of fetal congenital heart defects: Ventricular Septal Defect (VSD)
Source: PLoS One. 2019 Mar 21;14(3):e0200229. doi: 10.1371/journal.pone.0200229 (PMC6428297; doi:10.1371/journal.pone.0200229)

Sl No.	TargetID	position	Correlation coefficient	p-value	q-value	Exp mean	Meth mean
1	cg01554580	47324306	-2.85E-01	3.45E-06	4.30E-07	8.67	0.02
2	cg06868247	74960515	-2.33E-01	1.76E-03	6.75E-04	8.59	0.02
3	cg13537283	60609563	-0.2094771	9.14E-02	1.04E-02	9.31	0.02
4	cg23108125	176882586	-0.2108966	8.92E-02	1.02E-02	3.57	0.06
5	cg13790909	36391442	-0.251398	9.77E-06	1.24E-06	6.03	0.02
6	cg14103143	46969973	-0.2359481	2.80E-02	3.08E-03	10.19	0.02
7	cg19526908	49058333	-0.3800211	3.12E-04	7.89E-05	10.00	0.02
8	cg19679633	2752278	-0.3348457	1.12E-02	2.35E-03	2.66	0.06
9	cg24050613	186648279	-3.61E-01	2.33E-10	7.33E-11	11.08	0.07
10	cg27619163	7982806	-2.05E-01	3.86E-05	3.68E-06	1.45	0.09
11	cg02044989	25228615	-0.2177767	3.47E-03	8.86E-04	11.62	0.03
12	cg03119028	89458849	-0.1950511	3.76E-06	2.43E-07	8.79	0.13
13	cg04364339	91087703	-2.53E-01	5.59E-11	7.05E-12	9.63	0.08
14	cg04678743	130353515	-0.2923104	1.44E-02	3.63E-03	10.45	0.02
15	cg08974966	3078805	-2.70E-01	3.81E-04	3.20E-04	9.29	0.06
16	cg12207922	74063060	-0.2130291	4.24E-07	2.94E-08	10.14	0.01
17	cg12903924	242448061	-0.3441441	4.05E-02	8.92E-03	11.06	0.02
18	cg14957718	50243260	-2.23E-01	4.72E-06	5.12E-07	2.69	0.15
19	cg24429881	166809343	-4.12E-01	6.88E-11	1.72E-11	13.04	0.02
20	cg26767214	40762864	-2.58E-01	2.11E-02	3.17E-03	9.97	0.04
21	cg02825052	85753082	-3.07E-01	1.65E-10	3.91E-11	11.94	0.10
22	cg03228804	86589205	-0.236879	7.10E-02	8.42E-03	0.26	0.05
23	cg07387607	31587714	-2.91E-01	3.25E-09	4.83E-10	12.61	0.22
24	cg07557796	18690791	-4.01E-01	2.75E-11	7.94E-12	10.44	0.10
25	cg09904383	141031125	-2.81E-01	1.58E-04	2.21E-05	9.06	0.02
26	cg10241701	117113180	-4.45E-01	1.23E-12	4.21E-13	10.81	0.02
27	cg12614213	38324613	-2.45E-01	8.33E-05	1.78E-05	9.74	0.09
28	cg14312359	44113481	-0.256013	8.25E-02	9.73E-03	8.88	0.01
29	cg16015423	15217974	-5.20E-01	0.00E+00	0.00E+00	8.28	0.07
30	cg17949256	27507578	-3.50E-01	1.29E-05	2.29E-06	10.28	0.04
31	cg23340613	37311651	-2.77E-01	1.58E-10	2.71E-11	9.15	0.02

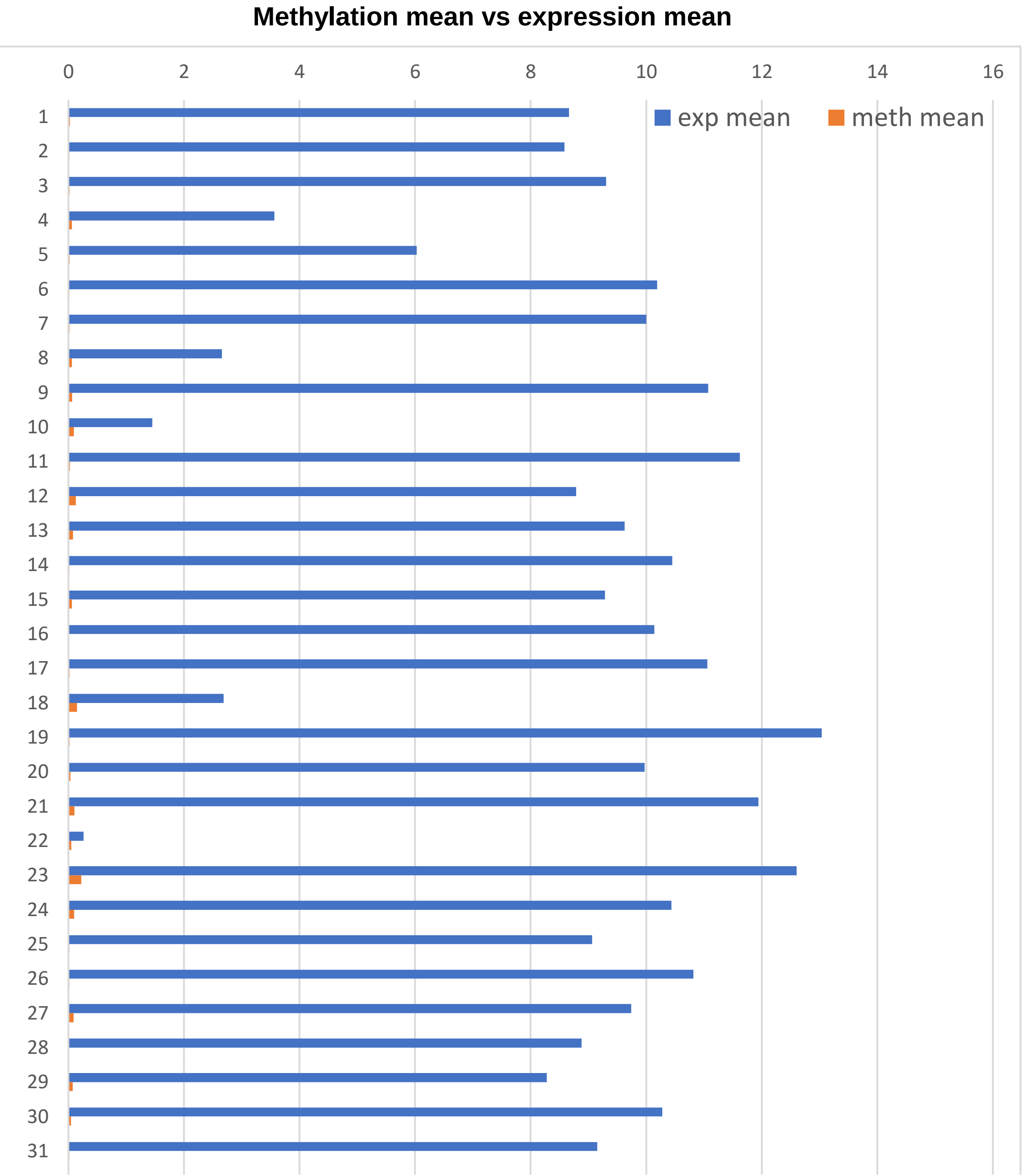

32	cg24398318	89205684	-0.4855648	7.57E-06	5.32E-06	8.38	0.03
33	cg24631360	111698088	-3.34E-01	2.60E-03	5.65E-04	8.34	0.02
34	cg01924596	31854504	-4.36E-01	1.06E-13	4.08E-14	7.79	0.23
35	cg04408595	71802608	-0.7180773	0.00E+00	0.00E+00	3.18	0.34
36	cg05728201	69215753	-5.85E-01	1.26E-22	1.77E-22	12.75	0.02
37	cg09409435	70099608	-0.3300146	3.11E-03	7.80E-04	7.65	0.02
38	cg15790941	39640517	-3.28E-01	1.06E-03	2.42E-04	8.62	0.02
39	cg17074816	68410573	-2.50E-01	1.02E-08	9.45E-10	7.86	0.06
40	cg18280382	126225996	-0.4394562	0.00E+00	0.00E+00	8.83	0.46
41	cg21682474	33112475	-0.4368215	2.09E-03	1.35E-03	9.52	0.04
42	cg22019999	41634387	-3.04E-01	2.32E-15	3.88E-16	8.19	0.01
43	cg22605179	29664171	-0.2249839	8.79E-10	8.40E-11	8.35	0.06
44	cg27594176	1619080	-2.34E-01	4.52E-06	9.92E-07	6.47	0.03
45	cg00125159	228290140	-3.73E-01	0.00E+00	0.00E+00	8.53	0.03
46	cg00310855	64879153	-0.3120763	3.38E-03	5.35E-04	11.05	0.05
47	cg02006257	153450999	-0.2328415	2.04E-03	3.35E-04	4.68	0.04
48	cg02376269	43398346	-1.98E-01	5.82E-06	7.95E-07	9.54	0.03
49	cg05315670	64037304	-0.407225	3.24E-02	2.34E-02	10.41	0.02
50	cg05986044	9614578	-3.88E-01	1.06E-09	2.18E-10	11.29	0.04
51	cg06516445	102963032	-2.52E-01	3.97E-03	6.82E-04	9.66	0.02
52	cg07000467	39424930	-2.74E-01	7.15E-04	9.33E-05	9.71	0.03
53	cg07435294	65554356	-0.2625926	1.34E-06	1.39E-07	1.65	0.27
54	cg07675334	29819451	-2.24E-01	2.83E-07	2.32E-08	12.00	0.15
55	cg09990790	73934953	-0.2421561	3.35E-11	3.52E-12	8.63	0.04
56	cg10106505	82777881	-0.48571	0.355556	0.06101	3.31	0.07
57	cg13139203	63581631	-3.60E-01	7.71E-13	4.30E-13	8.98	0.06
58	cg13692001	40023494	-0.2411424	2.47E-02	2.77E-03	5.61	0.02
59	cg14227032	95526284	-2.30E-01	4.01E-02	5.38E-03	10.17	0.02
60	cg14452706	103570781	-1.81E-01	5.26E-02	1.96E-02	1.57	0.15
61	cg15693066	66824189	-5.65E-01	2.05E-44	8.03E-45	2.52	0.66
62	cg18043157	150390308	-0.4781155	4.75E-21	1.39E-21	3.01	0.12
63	cg19416088	30162989	-0.4359736	2.94E-05	1.18E-05	8.03	0.02
64	cg21484586	35531859	-3.09E-01	4.14E-03	1.27E-03	6.17	0.05
65	cg22669060	42543530	-0.2068042	9.71E-08	5.83E-09	4.05	0.08

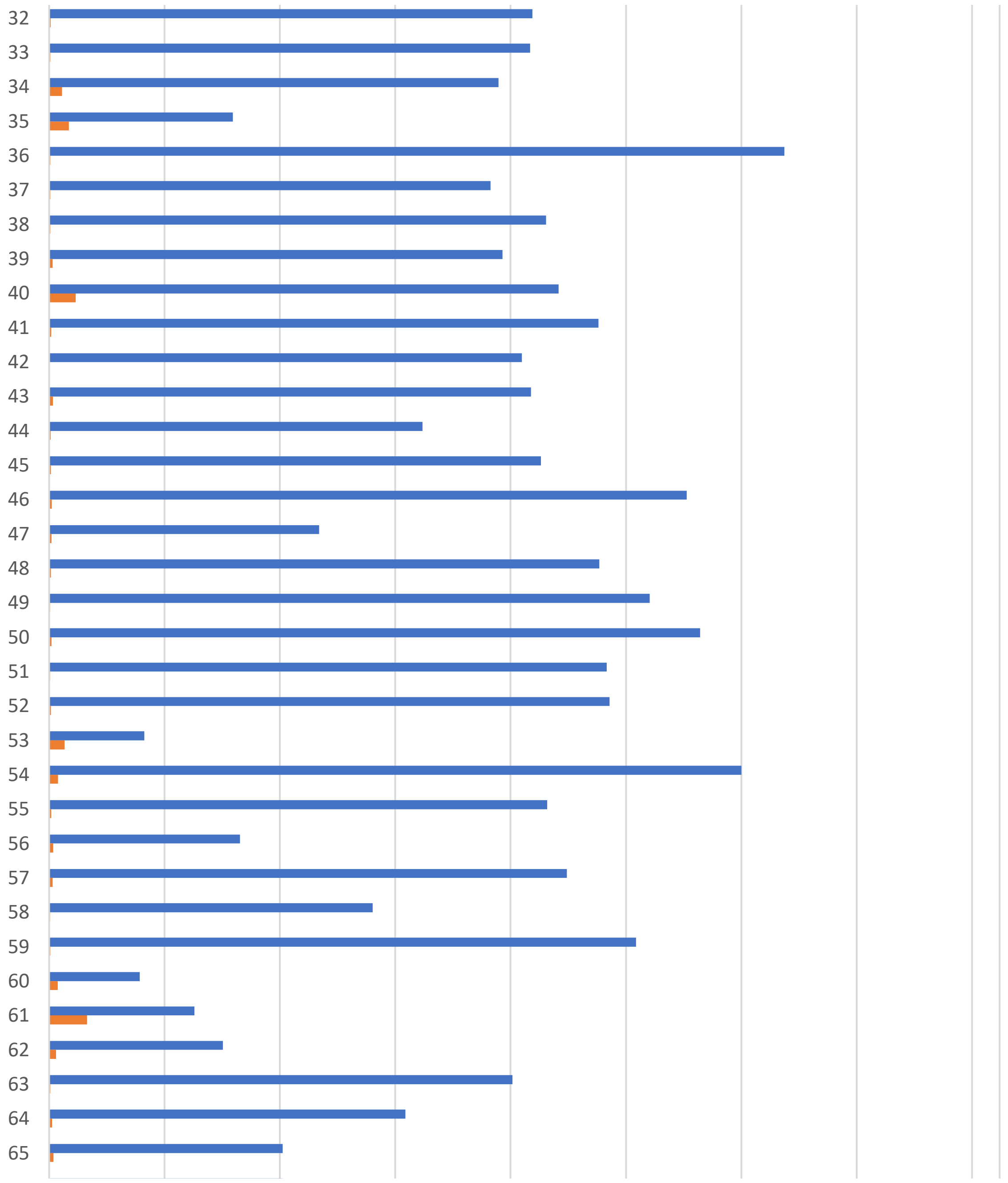

66	cg23003500	42543530	-0.2068042	9.71E-08	5.83E-09	4.05	0.08
67	cg23623270	42402962	-2.04E-01	3.18E-06	4.45E-07	10.32	0.36
68	cg23675362	180601130	-0.2927565	6.81E-16	9.95E-17	10.20	0.03
69	cg24780865	28025299	-3.11E-01	1.14E-04	1.72E-05	9.13	0.03
70	cg25335229	52643207	-3.94E-01	5.19E-10	1.12E-10	9.55	0.04
71	cg27123975	57697125	-1.96E-01	4.38E-05	4.56E-06	12.84	0.04
72	cg27589366	7745726	-0.3270941	2.09E-03	3.61E-04	9.49	0.02
73	cg03372099	73309462	-2.54E-01	3.90E-05	4.15E-06	7.29	0.03
74	cg04600122	39341829	-0.4700129	4.19E-03	1.93E-03	12.34	0.04
75	cg05180771	6576736	-0.3216157	8.72E-03	1.66E-03	10.54	0.01
76	cg07772605	195384752	-0.4651223	4.65E-03	2.07E-03	7.89	0.06
77	cg07807690	41157266	-2.70E-01	8.95E-10	2.24E-10	5.42	0.04
78	cg09163958	213123886	-3.56E-01	6.91E-09	2.64E-09	3.65	0.04
79	cg10347032	183543704	-2.49E-01	1.11E-03	8.06E-04	6.60	0.15
80	cg10366093	57414457	-0.3609422	1.21E-02	5.38E-03	4.19	0.57
81	cg12323089	89011145	-0.2106692	5.03E-02	5.05E-03	8.67	0.02
82	cg13771733	131591782	-2.33E-01	1.77E-03	1.95E-04	8.93	0.02
83	cg14673384	118992278	-0.3100094	1.16E-02	2.04E-03	8.28	0.01
84	cg15399923	209130889	-2.27E-01	4.31E-02	5.71E-03	8.97	0.02
85	cg16929354	34915386	-0.3071929	1.71E-13	1.99E-14	12.89	0.02
86	cg17973565	153606594	-0.2330685	3.00E-02	3.26E-03	10.05	0.02
87	cg18662566	128643056	-3.50E-01	4.53E-08	7.34E-09	11.56	0.02
88	cg18683875	49952384	-0.71429	0.136111	0.03084	4.58	0.02
89	cg19112957	47736463	-2.67E-01	3.26E-03	5.25E-04	5.03	0.02
90	cg19244640	35325348	-0.3106115	9.12E-03	2.50E-03	11.92	0.02
91	cg22865402	186344630	-0.3683788	9.03E-04	2.75E-04	9.69	0.02
92	cg22903286	77564084	-3.06E-01	6.02E-03	1.13E-03	9.28	0.01
93	cg23029546	155294555	-0.7383689	1.31E-05	2.37E-04	8.53	0.16
94	cg23255774	57791959	-0.2303711	3.20E-02	3.44E-03	5.11	0.04
95	cg25324601	104942705	-0.77143	0.102778	0.02632	3.52	0.03
96	cg26674752	43634212	-0.1303559	8.20E-02	1.38E-02	9.01	0.10
97	cg27545919	37157995	-0.668515	4.21E-52	2.03E-52	5.36	0.28
98	cg01573747	17008740	-3.28E-01	7.82E-09	1.99E-09	4.26	0.41
99	cg03182819	63053327	-4.44E-01	1.45E-12	4.89E-13	13.65	0.02

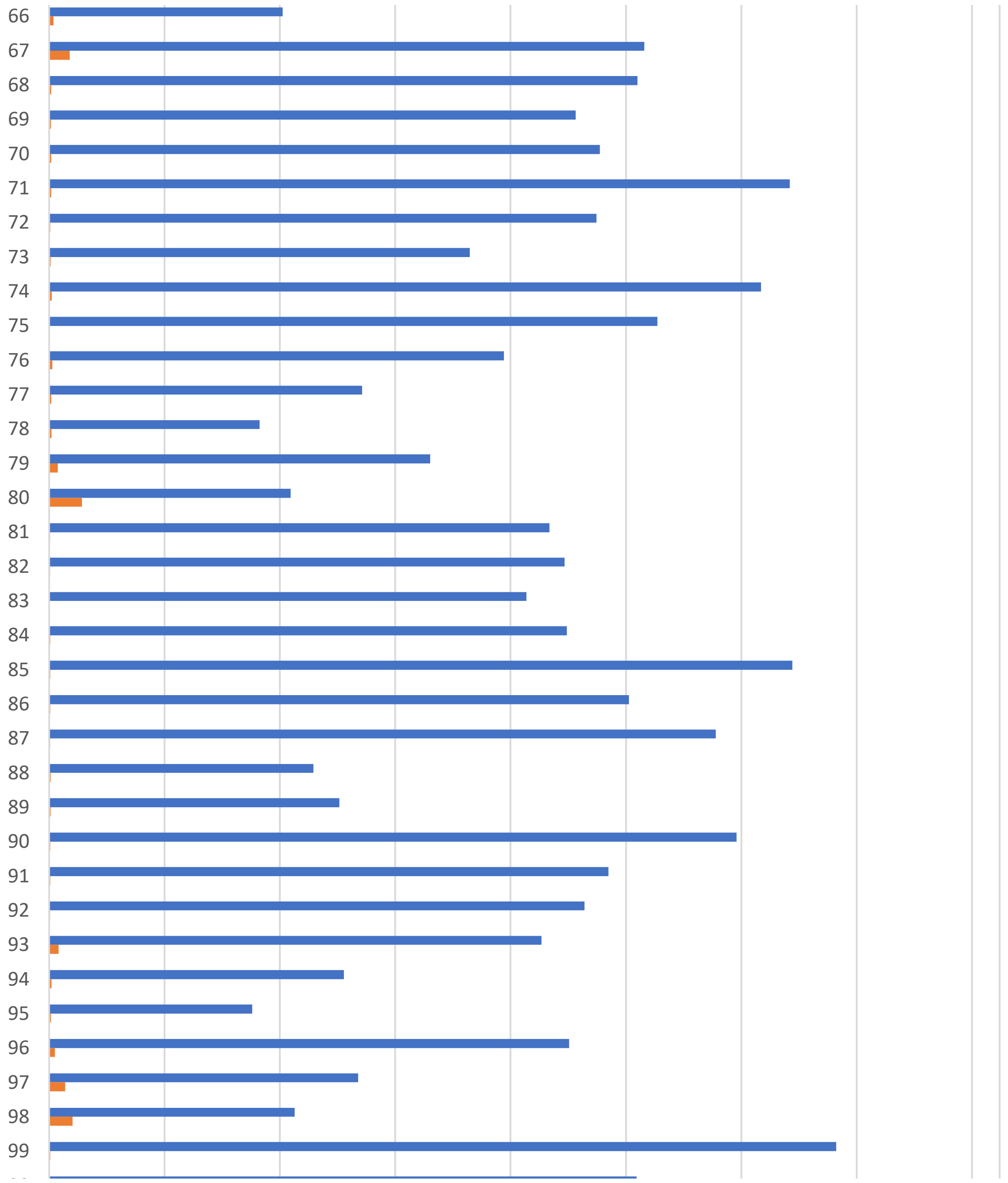

100	cg03205007	55845736	-3.92E-01	2.24E-09	1.24E-09	10.19	0.14
101	cg04039414	27280154	-2.12E-01	1.00E-05	1.14E-06	10.38	0.03
102	cg05367248	13203479	-0.533109	1.22E-04	1.53E-04	8.67	0.02
103	cg06154313	95360494	-0.431306	0.00E+00	0.00E+00	7.04	0.54
104	cg06622999	72666281	-2.75E-01	1.64E-03	3.59E-04	3.27	0.09
105	cg06705366	74162621	-0.4819931	3.08E-06	1.85E-06	5.60	0.19
106	cg07448795	21368419	-2.51E-01	5.01E-04	1.05E-04	1.30	0.10
107	cg08904082	60457901	-3.98E-01	2.02E-16	1.55E-16	10.75	0.03
108	cg09295695	31649093	-0.558666	1.52E-07	2.25E-07	3.52	0.39
109	cg10088527	74609893	-0.82857	0.058333	0.02023	3.28	0.02
110	cg10802132	178487500	-0.4195099	0.00E+00	0.00E+00	5.09	0.13
111	cg11309039	133702676	-0.236036	1.65E-01	2.44E-02	4.65	0.04
112	cg11891983	45272974	-0.4562905	1.42E-03	4.90E-04	11.85	0.03
113	cg13320518	114196999	-0.2865402	5.12E-02	6.80E-03	8.13	0.02
114	cg13667676	58239952	-0.3205494	7.05E-03	2.03E-03	14.99	0.02
115	cg13674316	55518174	-0.4410551	1.88E-03	1.24E-03	11.42	0.03
116	cg14258555	50180725	-2.18E-01	9.79E-07	6.90E-08	11.20	0.02
117	cg15066100	17413959	-0.4933112	1.68E-06	1.13E-06	8.59	0.03
118	cg15637465	168728270	-3.10E-01	2.01E-03	4.12E-04	7.12	0.53
119	cg16076651	52930071	-0.3329569	3.31E-09	7.06E-10	9.56	0.03
120	cg16127594	95393115	-3.86E-01	0.00E+00	0.00E+00	10.99	0.29
121	cg16277479	13782796	-0.3007632	4.04E-02	5.67E-03	5.56	0.06
122	cg16727585	109592662	-3.33E-01	2.15E-07	3.18E-08	11.38	0.05
123	cg16884841	1983925	-0.65714	0.175	0.03652	2.32	0.02
124	cg17284070	473007	-0.1829034	1.44E-02	3.01E-03	8.66	0.01
125	cg17679824	65018933	-1.95E-01	8.64E-06	6.22E-07	9.41	0.08
126	cg19323951	64993076	-0.2762838	2.09E-02	4.95E-03	8.64	0.10
127	cg19342764	53935265	-0.2742943	5.81E-14	7.41E-15	8.83	0.03
128	cg20872579	6643098	-2.16E-01	4.85E-03	2.84E-03	10.76	0.01
129	cg21274857	57669179	-4.52E-01	9.19E-09	2.66E-09	9.65	0.07
130	cg21509931	7218030	-2.64E-01	2.33E-09	5.61E-10	10.16	0.06
131	cg21685750	37193358	-2.11E-01	1.43E-06	2.06E-07	7.81	0.03
132	cg22137448	2258989	-0.54286	0.297222	0.0535	6.70	0.04
133	cg24973420	6762535	-0.2	0.713889	0.11203	4.47	0.03

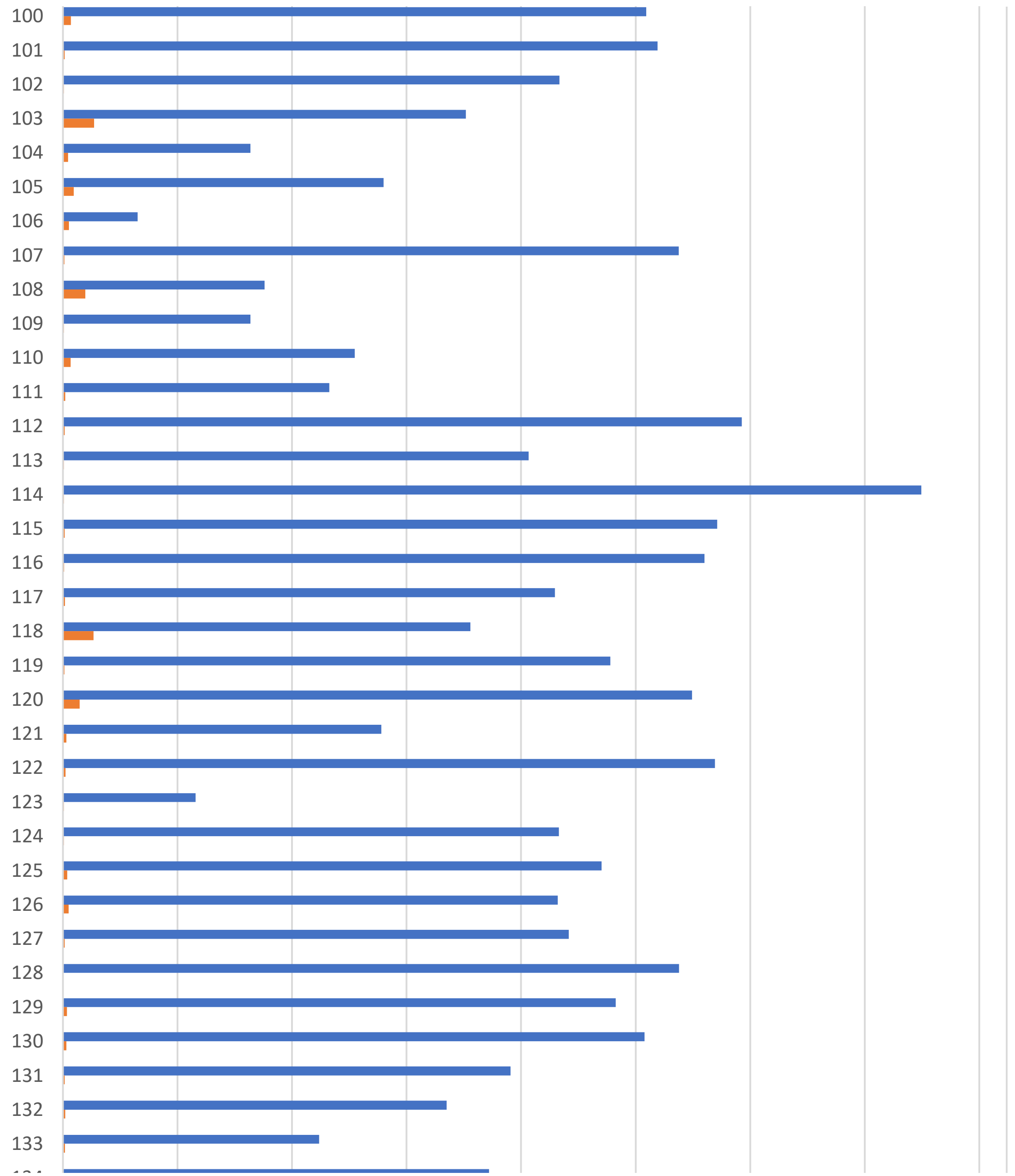

134	cg26214747	111497064	-0.2194251	1.86E-07	1.33E-08	7.44	0.08
135	cg26342670	56320924	-0.3549391	7.03E-03	1.33E-03	9.23	0.02
136	cg26699183	32581556	-0.1303726	8.20E-02	1.38E-02	11.15	0.01
137	cg27626746	126081776	-4.59E-01	1.54E-10	2.87E-10	8.36	0.02
138	cg00531823	78089769	-0.82857	0.058333	0.02023	4.37	0.03
139	cg01022678	52095538	-1.72E-01	2.69E-02	1.15E-02	3.95	0.02
140	cg02075087	184426383	-0.1993053	7.57E-03	1.73E-03	7.04	0.02
141	cg02367949	94964516	-0.5062945	6.60E-03	8.45E-03	6.10	0.05
142	cg03771121	45430052	-0.5488493	7.08E-05	1.03E-04	8.95	0.02
143	cg03899775	45430052	-0.5488493	7.08E-05	1.03E-04	8.95	0.02
144	cg03993926	105948416	-5.49E-01	1.44E-19	1.44E-19	9.02	0.02
145	cg04406910	31926623	-1.91E-01	1.80E-05	2.91E-06	10.45	0.04
146	cg05027081	91739204	-9.14E-02	3.24E-02	1.54E-02	6.79	0.03
147	cg05597554	46038994	-0.4423	2.658E-21	1.195E-21	12.15	0.02
148	cg06914050	56119237	-1.89E-01	2.54E-03	3.96E-04	11.46	0.03
149	cg07305215	5296031	-0.2716707	5.05E-08	9.10E-09	8.10	0.03
150	cg08981282	2653240	-0.3804768	3.07E-04	7.78E-05	5.24	0.07
151	cg09874822	1203997	-4.40E-01	1.17E-09	4.88E-10	9.52	0.33
152	cg11684022	247495769	-0.1953328	3.64E-06	2.36E-07	8.61	0.05
153	cg11737710	37959864	-0.3602646	6.58E-04	1.43E-04	5.65	0.04
154	cg11762839	17565885	-2.31E-01	7.37E-04	1.31E-04	3.24	0.05
155	cg12597276	121297486	-4.42E-01	3.76E-09	2.57E-09	2.01	0.02
156	cg13137476	183580694	-0.2399407	5.88E-11	6.09E-12	11.51	0.03
157	cg13227621	45671361	-3.98E-01	3.58E-10	7.94E-11	10.22	0.07
158	cg13462232	17551166	-2.88E-01	7.94E-05	2.19E-05	12.72	0.05
159	cg13595191	39715747	-2.79E-01	5.27E-08	6.87E-09	14.85	0.02
160	cg13944175	52222698	-2.13E-01	1.04E-04	1.51E-05	1.25	0.65
161	cg14001567	86974838	-2.51E-01	9.92E-04	7.31E-04	5.62	0.03
162	cg14428530	72874528	-0.2711361	1.15E-13	1.43E-14	9.79	0.08
163	cg15463775	93544534	-3.14E-01	6.31E-10	2.44E-10	8.78	0.04
164	cg15946310	159436034	-2.14E-01	2.95E-05	5.67E-06	9.91	0.02
165	cg16054451	47755353	-2.46E-01	9.70E-04	1.13E-04	10.08	0.05
166	cg17114847	47736795	-4.55E-01	3.17E-27	6.00E-28	2.10	0.24
167	cg17206034	38206839	-1.69E-01	2.81E-02	1.18E-02	10.44	0.01

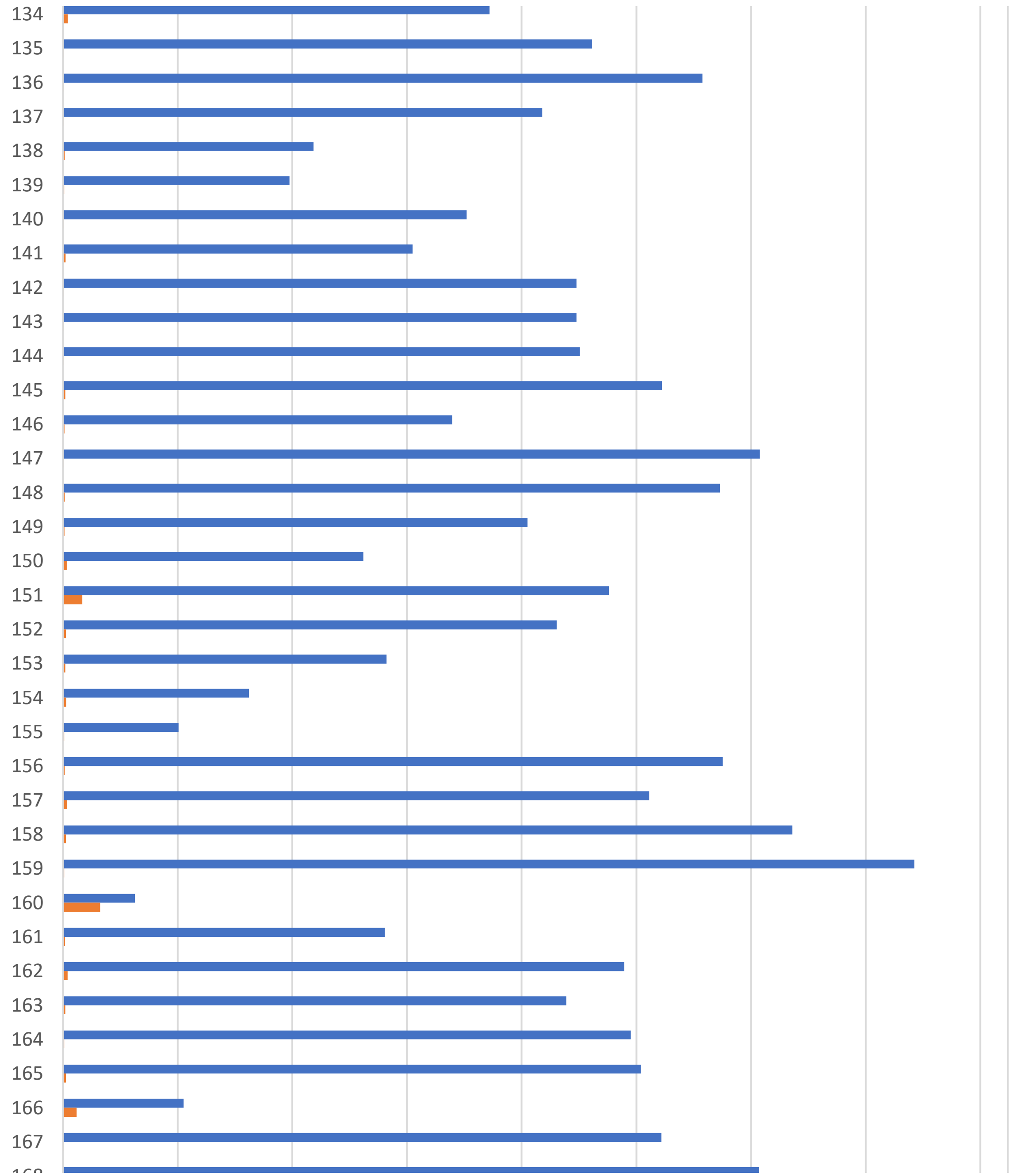

168	cg18338863	114178495	-0.4354451	3.01E-05	1.20E-05	12.14	0.03
169	cg19697239	752646	-0.3755923	3.71E-04	9.05E-05	5.95	0.45
170	cg20451226	44488181	-0.4691296	0.00E+00	0.00E+00	6.42	0.09
171	cg20810288	19638859	-2.11E-01	1.95E-06	3.53E-07	8.63	0.03
172	cg22994808	33146521	-0.3048714	1.15E-01	5.60E-02	9.32	0.21
173	cg23661343	616707	-0.2350064	1.67E-01	2.47E-02	2.70	0.13
174	cg24310460	33055971	-0.77143	0.102778	0.02632	4.94	0.02
175	cg25012434	128842125	-3.43E-01	3.77E-05	1.31E-05	11.24	0.04
176	cg25532501	134201505	-0.465598	0.00E+00	0.00E+00	7.77	0.05
177	cg26182254	57210406	-0.7044221	0.00E+00	0.00E+00	5.02	0.39
178	cg26658439	20817948	-1.56E-01	4.28E-02	1.66E-02	9.06	0.03
179	cg26894438	15902633	-0.3833345	0.00E+00	0.00E+00	8.03	0.03
180	cg27217916	85843214	-0.2282865	4.92E-10	4.77E-11	10.35	0.01

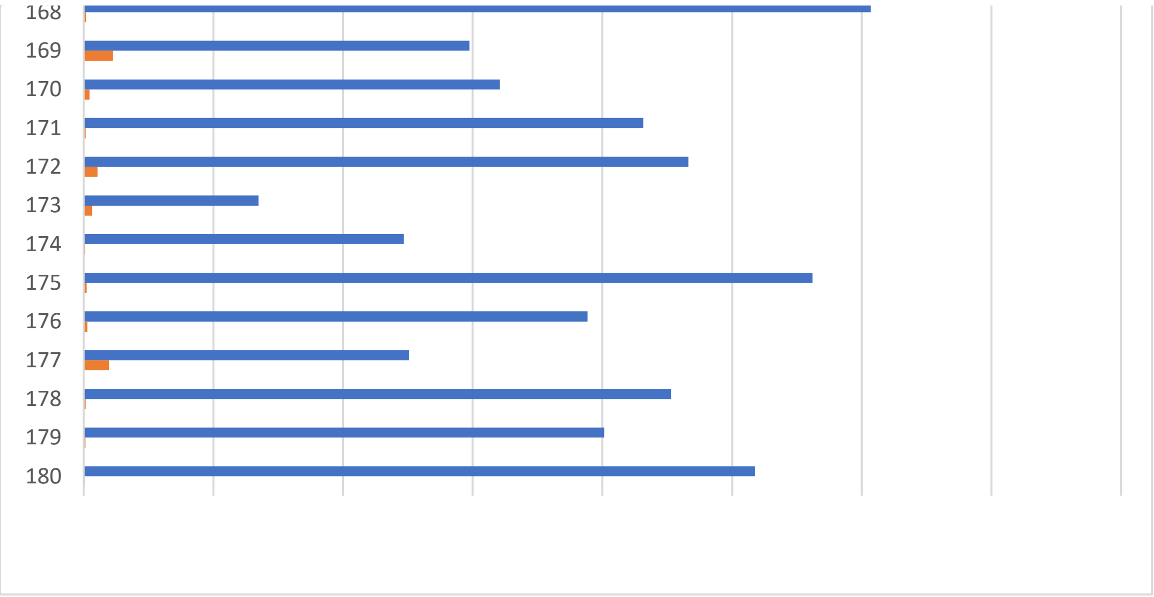

Supplement: S8 Table — 180 differentially methylated CpG targets were correlated with expression (RNA-seq) data. A bar chart was generated for each CpG target showing the proportion of methylation and mean of expression of the gene in which the CpG target resided. (PDF) [file pone.0200229.s011.pdf]
