# Supplementary material for: Placental epigenetics for evaluation of fetal congenital heart defects: Ventricular Septal Defect (VSD)
Source: PLoS One. 2019 Mar 21;14(3):e0200229. doi: 10.1371/journal.pone.0200229 (PMC6428297; doi:10.1371/journal.pone.0200229)
Supplement: S9 Table — ENCODE data showing the H3K27Ac layering on each CpG site presenting an open chromatin conformation. These CpG targets were also occupied with various transcription initiation factors, mostly PolR2A. The position of each CpG site was also noted in respect to the gene in which it resided. Some of the differentially methylated CpG sites that were resided in intronic or 1st exonic regions, signifying their essential function in modulating transcription. (PDF) [file pone.0200229.s012.pdf]

Target ID	Genes	Position	Correlation coefficient	p-value	q-value	Expression mean	Methylation mean	H3k27Ac	Transcription factors	Location
cg14001567	TP53TG1; CROT	60609563	-0.2094771	9.14E-02	1.04E-02	9.31	0.02	moderate	polr2a	promoter
cg07387607	BAT2	47324306	-2.85E-01	3.45E-06	4.30E-07	8.67	0.02	high	polr2a	1st exonic non-coding
cg06622999	TRHDE	74960515	-2.33E-01	1.76E-03	6.75E-04	8.59	0.02	high	polr2a	exonic
cg13944175	HAS1	176882586	-0.2108966	8.92E-02	1.02E-02	3.57	0.06	moderate	polr2a,myc	1st exonic non-coding
cg27619163	ALOX12B	7982806	-2.05E-01	3.86E-05	3.68E-06	1.45	0.09	low	ctcf	exonic
cg14957718	SLC38A3	46969973	-0.2359481	2.80E-02	3.08E-03	10.19	0.02	low	sp1	promoter
cg23661343	CLUL1	2752278	-0.3348457	1.12E-02	2.35E-03	2.66	0.06	moderate	none	1st exonic non-coding
cg07675334	MAZ	49058333	-0.3800211	3.12E-04	7.89E-05	10.00	0.02	high	polr2a	1st exonic non-coding
cg26674752	CSGALNACT2	36391442	-0.251398	9.77E-06	1.24E-06	6.03	0.02	moderate	polr2a	1st exonic non-coding
cg17114847	AGBL2	186648279	-3.61E-01	2.33E-10	7.33E-11	11.08	0.07	moderate	polr2a	promoter
cg09874822	CACNA1H	3078805	-2.70E-01	3.81E-04	3.20E-04	9.29	0.06	low	nr3c1,polr2a	promoter
cg22669060	IFNGR2	89458849	-0.1950511	3.76E-06	2.43E-07	8.79	0.13	high	polr2a	promoter
cg02825052	PICALM	40762864	-2.58E-01	2.11E-02	3.17E-03	9.97	0.04	moderate	none	promoter
cg13320518	VTI1A; ZDHHC6	74063060	-0.2130291	4.24E-07	2.94E-08	10.14	0.01	moderate	polr2a	1st exonic non-coding
cg21484586	HPN	91087703	-2.53E-01	5.59E-11	7.05E-12	9.63	0.08	high	polr2a	1st exonic non-coding
cg06154313	RBP4	166809343	-4.12E-01	6.88E-11	1.72E-11	13.04	0.02	low	ctcf	intronic
cg09295695	LY6G5C	25228615	-0.2177767	3.47E-03	8.86E-04	11.62	0.03	low	ctcf	promoter
cg03205007	SMEK2	50243260	-2.23E-01	4.72E-06	5.12E-07	2.69	0.15	none	none	1stintronic
cg10366093	YPEL4	242448061	-0.3441441	4.05E-02	8.92E-03	11.06	0.02	low	polr2a	1st exonic non-coding
cg00310855	C11orf2; TM7SF2	130353515	-0.2923104	1.44E-02	3.63E-03	10.45	0.02	moderate	polr2a	1st exonic non-coding
cg16884841	DCP1B	111698088	-3.34E-01	2.60E-03	5.65E-04	8.34	0.02	low	e2f1,polr2a	1stintronic
cg22994808	MAP1LC3A	31587714	-2.91E-01	3.25E-09	4.83E-10	12.61	0.22	high	polr2a,nfya	intronic
cg03771121	CARHSP1	37311651	-2.77E-01	1.58E-10	2.71E-11	9.15	0.02	moderate	polr2a,cebpb	1st exonic non-coding
cg08904082	C5orf43	117113180	-4.45E-01	1.23E-12	4.21E-13	10.81	0.02	high	polr2a,ctcf	1stintronic
cg21274857	CGNL1	141031125	-2.81E-01	1.58E-04	2.21E-05	9.06	0.02	none	polr2a	promoter
cg09904383	FCHSD1	38324613	-2.45E-01	8.33E-05	1.78E-05	9.74	0.09	high	none	1st exonic non-coding

cg15463775	MTF2	86589205	-0.236879	7.10E-02	8.42E-03	0.26	0.05	moderate	none	exonic
cg00125159	C1orf35	27507578	-3.50E-01	1.29E-05	2.29E-06	10.28	0.04	low	e2f1,polr2a,ctcf	promoter
cg03119028	CCBL2; RBMXL1	85753082	-3.07E-01	1.65E-10	3.91E-11	11.94	0.10	moderate	ctcf,polr2a	1stintronic
cg19244640	C1orf212	89205684	-0.4855648	7.57E-06	5.32E-06	8.38	0.03	moderate	polr2a	1st exonic non-coding
cg02376269	UBR1	18690791	-4.01E-01	2.75E-11	7.94E-12	10.44	0.10	low	none	exonic
cg27594176	C17orf91	44113481	-0.256013	8.25E-02	9.73E-03	8.88	0.01	none	none	promoter
cg16054451	SPOP	15217974	-5.20E-01	0.00E+00	0.00E+00	8.28	0.07	low	none	promoter
cg14103143	ATP5G1	68410573	-2.50E-01	1.02E-08	9.45E-10	7.86	0.06	high	none	promoter
cg23108125	PRR7	71802608	-0.7180773	0.00E+00	0.00E+00	3.18	0.34	none	ctcf	1st exonic non-coding
cg22605179	EWSR1	29664171	-0.2249839	8.79E-10	8.40E-11	8.35	0.06	high	polr2a	1st exonic non-coding
cg03228804	MTHFSD; FLJ30679	33112475	-0.4368215	2.09E-03	1.35E-03	9.52	0.04	high	polr2a	1stintronic
cg01924596	PDZD2	70099608	-0.3300146	3.11E-03	7.80E-04	7.65	0.02	moderate	polr2a	1st exonic non-coding
cg18043157	ULBP3	31854504	-4.36E-01	1.06E-13	4.08E-14	7.79	0.23	low	polr2a	intronic
cg17973565	C1orf77; S100A13	126225996	-0.4394562	0.00E+00	0.00E+00	8.83	0.46	none	none	1st exonic non-coding
cg05027081	CDC7	41634387	-3.04E-01	2.32E-15	3.88E-16	8.19	0.01	moderate	polr2a	intergenic
cg08974966	CARS	69215753	-5.85E-01	1.26E-22	1.77E-22	12.75	0.02	moderate	polr2a,ctcf	1st exonic non-coding
cg08981282	LOC652276	64037304	-0.407225	3.24E-02	2.34E-02	10.41	0.02	high	polr2a	cebpb
cg23340613	CCDC75; HEATR5B	95526284	-2.30E-01	4.01E-02	5.38E-03	10.17	0.02	low	none	1stintronic
cg02006257	RGS17	64879153	-0.3120763	3.38E-03	5.35E-04	11.05	0.05	none	nfya	1st exonic non-coding
cg09990790	COX18	57697125	-1.96E-01	4.38E-05	4.56E-06	12.84	0.04	high	polr2a,sp1	promoter
cg25532501	GLB1L2	73934953	-0.2421561	3.35E-11	3.52E-12	8.63	0.04	low	polr2a	1stintronic
cg07435294	OVOL1	39424930	-2.74E-01	7.15E-04	9.33E-05	9.71	0.03	high	polr2a	1st exonic non-coding
cg14258555	PRMT1	102963032	-2.52E-01	3.97E-03	6.82E-04	9.66	0.02	moderate	polr2a	promoter
cg04364339	IFIT3	40023494	-0.2411424	2.47E-02	2.77E-03	5.61	0.02	moderate	polr2a,ctcf	1st exonic non-coding
cg15066100	ABHD8	35531859	-3.09E-01	4.14E-03	1.27E-03	6.17	0.05	none	polr2a	intronic
cg17284070	LOC25845	9614578	-3.88E-01	1.06E-09	2.18E-10	11.29	0.04	none	polr2a	promoter
cg24780865	MTIF3	42543530	-0.2068042	9.71E-08	5.83E-09	4.05	0.08	low	fos	1stintronic

cg12207922	GFM2	29819451	-2.24E-01	2.83E-07	2.32E-08	12.00	0.15	moderate	polr2a,ctcf	intronic
cg04678743	TSGA13; COPG2	28025299	-3.11E-01	1.14E-04	1.72E-05	9.13	0.03	moderate	none	intergenic
cg04408595	EIF4E3;GPR27	65554356	-0.2625926	1.34E-06	1.39E-07	1.65	0.27	low	none	promoter
cg27123975	CLTC	153450999	-0.2328415	2.04E-03	3.35E-04	4.68	0.04	none	none	1stinronic
cg26767214	CHST14	66824189	-5.65E-01	2.05E-44	8.03E-45	2.52	0.66	moderate	none	promoter
cg19526908	NDUFAF3; MIR191	42402962	-2.04E-01	3.18E-06	4.45E-07	10.32	0.36	moderate	polr2a	promoter
cg13667676	CTDSP2	82777881	-0.48571	0.355556	0.06101	3.31	0.07	none	none	intronic
cg27545919	ZNF461	7745726	-0.3270941	2.09E-03	3.61E-04	9.49	0.02	low	ctcf,polr2a	1st exonic non-coding
cg21682474	N4BP2L2	43398346	-1.98E-01	5.82E-06	7.95E-07	9.54	0.03	moderate	polr2a	promoter
cg06705366	FAM169A	30162989	-0.4359736	2.94E-05	1.18E-05	8.03	0.02	low	polr2a	promoter
cg20872579	MRFAP1	150390308	-0.4781155	4.75E-21	1.39E-21	3.01	0.12	none	polr2a	promoter
cg26214747	NCRNA00219; SNORA13	42543530	-0.2068042	9.71E-08	5.83E-09	4.05	0.08	none	none	1stintronic
cg12903924	STK25	180601130	-0.2927565	6.81E-16	9.95E-17	10.20	0.03	none	polr2a	promoter
cg23029546	RUSC1	128643056	-3.50E-01	4.53E-08	7.34E-09	11.56	0.02	none	ctcf	1stintronic
cg10106505	TAF1C	153606594	-0.2330685	3.00E-02	3.26E-03	10.05	0.02	high	polr2a	1st exonic non-coding
cg11684022	ZNF496	43634212	-0.1303559	8.20E-02	1.38E-02	9.01	0.10	high	polr2a	promoter
cg27589366	TRAPPC5	73309462	-2.54E-01	3.90E-05	4.15E-06	7.29	0.03	low	polr2a	promoter
cg24050613	ST6GAL1	118992278	-0.3100094	1.16E-02	2.04E-03	8.28	0.01	low	sp1,polr2a,	1st exonic non-coding
cg01022678	ITGA1;PELO	39341829	-0.4700129	4.19E-03	1.93E-03	12.34	0.04	high	polr2a	promoter
cg13139203	C11orf84	77564084	-3.06E-01	6.02E-03	1.13E-03	9.28	0.01	high	polr2a,myc	promoter
cg10241701	CD58	6576736	-0.3216157	8.72E-03	1.66E-03	10.54	0.01	none	polr2a	promoter
cg15399923	PIKFYVE	183543704	-2.49E-01	1.11E-03	8.06E-04	6.60	0.15	none	none	promoter
cg24429881	POGK	89011145	-0.2106692	5.03E-02	5.05E-03	8.67	0.02	moderate	polr2a	exonic
cg18280382	ST3GAL4	41157266	-2.70E-01	8.95E-10	2.24E-10	5.42	0.04	moderate	polr2a	1st exonic non-coding
cg16277479	PDPN	104942705	-0.77143	0.102778	0.02632	3.52	0.03	none	none	intronic
cg14227032	BICD2	209130889	-2.27E-01	4.31E-02	5.71E-03	8.97	0.02	low	sp1,myc,polr2a	promoter
cg17206034	OXSR1	49952384	-0.71429	0.136111	0.03084	4.58	0.02	none	none	intronic

cg18683875	RBM6	155294555	-0.7383689	1.31E-05	2.37E-04	8.53	0.16	moderate	none	1st exonic non-coding
cg07557796	ROCK1	195384752	-0.4651223	4.65E-03	2.07E-03	7.89	0.06	moderate	polr2a	promoter
cg04600122	HNRNPL	34915386	-0.3071929	1.71E-13	1.99E-14	12.89	0.02	moderate	polr2a	1st exonic non-coding
cg14428530	SMC5	213123886	-3.56E-01	6.91E-09	2.64E-09	3.65	0.04	none	polr2a	promoter
cg19342764	ZNF761; LOC147804	57414457	-0.3609422	1.21E-02	5.38E-03	4.19	0.57	none	polr2a	exonic
cg26658439	LOC81691; ERI2	17413959	-0.4933112	1.68E-06	1.13E-06	8.59	0.03	low	polr2a	1st exonic non-coding
cg26182254	LOC145783; TCF12	109592662	-3.33E-01	2.15E-07	3.18E-08	11.38	0.05	moderate	none	intronic
cg11309039	CDKL3	133702676	-0.236036	1.65E-01	2.44E-02	4.65	0.04	moderate	ctcf,polr2a	promoter
cg05986044	IAH1	57669179	-4.52E-01	9.19E-09	2.66E-09	9.65	0.07	none	none	1stintronic
cg17074816	CENPC1	95393115	-3.86E-01	0.00E+00	0.00E+00	10.99	0.29	high	polr2a	promoter
cg02075087	ING2	17008740	-3.28E-01	7.82E-09	1.99E-09	4.26	0.41	low	none	exonic
cg15693066	RHOD	58239952	-0.3205494	7.05E-03	2.03E-03	14.99	0.02	high	polr2a	1st exonic non-coding
cg09163958	VASH2	1983925	-0.65714	0.175	0.03652	2.32	0.02	none	none	intronic
cg12323089	MRPL46; MRPS11	74162621	-0.4819931	3.08E-06	1.85E-06	5.60	0.19	none	none	promoter
cg09409435	PDXDC2	52930071	-0.3329569	3.31E-09	7.06E-10	9.56	0.03	low	polr2a,myc	promoter
cg16015423	SYDE1	63053327	-4.44E-01	1.45E-12	4.89E-13	13.65	0.02	high	polr2a	promoter
cg16076651	FBXO9	7218030	-2.64E-01	2.33E-09	5.61E-10	10.16	0.06	low	polr2a	1st exonic non-coding
cg00531823	SEPT11	74609893	-0.82857	0.058333	0.02023	3.28	0.02	none	none	promoter
cg23003500	VIPR1	31649093	-0.558666	1.52E-07	2.25E-07	3.52	0.39	none	none	intronic
cg01573747	CPAMD8	55518174	-0.4410551	1.88E-03	1.24E-03	11.42	0.03	moderate	polr2a,e2fa	promoter
cg07807690	NFYC	13203479	-0.533109	1.22E-04	1.53E-04	8.67	0.02	low	polr2a,e2f1	promoter
cg24973420	MRFAP1L1	6643098	-2.16E-01	4.85E-03	2.84E-03	10.76	0.01	moderate	polr2a	1stintronic
cg15946310	TTC1	6762535	-0.2	0.713889	0.11203	4.47	0.03	none	none	intronic
cg17679824	SGTB; NLN	27280154	-2.12E-01	1.00E-05	1.14E-06	10.38	0.03	moderate	polr2a,cebpb,nfya	promoter
cg18662566	AMMECR1L	21368419	-2.51E-01	5.01E-04	1.05E-04	1.30	0.10	moderate	polr2a	1st exonic non-coding
cg17949256	MYO18A	13782796	-0.3007632	4.04E-02	5.67E-03	5.56	0.06	none	none	intronic
cg03182819	GNA13	50180725	-2.18E-01	9.79E-07	6.90E-08	11.20	0.02	high	polr2a	1stintronic

cg01554580	KIF9	32581556	-0.1303726	8.20E-02	1.38E-02	11.15	0.01	high	polr2a	1st exonic non-coding
cg21685750	STRN	95360494	-0.431306	0.00E+00	0.00E+00	7.04	0.54	none	polr2a	intronic
cg13771733	C9orf114	2258989	-0.54286	0.297222	0.0535	6.70	0.04	none	none	1st exonic non-coding
cg26699183	RALY	45272974	-0.4562905	1.42E-03	4.90E-04	11.85	0.03	none	none	intergenic
cg16127594	CNN3	126081776	-4.59E-01	1.54E-10	2.87E-10	8.36	0.02	low	polr2a	1stintronic
cg12614213	FGFR1	65018933	-1.95E-01	8.64E-06	6.22E-07	9.41	0.08	low	polr2a	1stintronic
cg07448795	P2RX6	64993076	-0.2762838	2.09E-02	4.95E-03	8.64	0.10	moderate	ctcf	exonic
cg07772605	SDHAP2	168728270	-3.10E-01	2.01E-03	4.12E-04	7.12	0.53	none	none	promoter
cg13674316	MAPK1IP1L	55845736	-3.92E-01	2.24E-09	1.24E-09	10.19	0.14	high	none	promoter
cg14452706	C14orf73	37193358	-2.11E-01	1.43E-06	2.06E-07	7.81	0.03	low	none	1stintronic
cg03993926	KBTBD3; AASDHPPT	72666281	-2.75E-01	1.64E-03	3.59E-04	3.27	0.09	low	none	intronic
cg24631360	ABHD10	114196999	-0.2865402	5.12E-02	6.80E-03	8.13	0.02	none	none	intronic
cg20810288	PQLC2; AKR7A2	56320924	-0.3549391	7.03E-03	1.33E-03	9.23	0.02	low	polr2a	1st exonic non-coding
cg13790909	NFKBID	78089769	-0.82857	0.058333	0.02023	4.37	0.03	none	none	1st exonic non-coding
cg06868247	NPC2; ISCA2	47736795	-4.55E-01	3.17E-27	6.00E-28	2.10	0.24	moderate	polr2a	1st exonic non-coding
cg11762839	USH1C	1203997	-4.40E-01	1.17E-09	4.88E-10	9.52	0.33	high	none	exonic
cg27217916	USP39	45430052	-0.5488493	7.08E-05	1.03E-04	8.95	0.02	none	none	intergenic
cg03372099	FAM168A	91739204	-9.14E-02	3.24E-02	1.54E-02	-0.68	0.03	none	none	intronic
cg07000467	DAB2	616707	-0.2350064	1.67E-01	2.47E-02	2.70	0.13	moderate	polr2a	promoter
cg15637465	SLIT3	46038994	-0.4423	2.658E-21	1.195E-21	12.15	0.02	low	polr2a	promoter
cg27626746	RPUSD4; FAM118B	183580694	-0.2399407	5.88E-11	6.09E-12	11.51	0.03	high	e2f1,myc	promoter
cg14673384	HINFP	17551166	-2.88E-01	7.94E-05	2.19E-05	12.72	0.05	moderate	none	promoter
cg10347032	MAP6D1	134201505	-0.465598	0.00E+00	0.00E+00	7.77	0.05	none	none	istintronic
cg19416088	UCRC; ZMAT5	52222698	-2.13E-01	1.04E-04	1.51E-05	1.25	0.65	none	none	exonic
cg07305215	ZFP161	184426383	-0.1993053	7.57E-03	1.73E-03	7.04	0.02	low	none	exonic
cg20451226	ZNF155	52095538	-1.72E-01	2.69E-02	1.15E-02	3.95	0.02	high	polr2a	1st exonic non-coding

cg02367949	SESN3	105948416	-5.49E-01	1.44E-19	1.44E-19	9.02	0.02	high	polr2a	1st exonic non-coding
cg04039414	NSMCE1	45430052	-0.5488493	7.08E-05	1.03E-04	8.95	0.02	low	polr2a,myc	promoter
cg05597554	COG3	57210406	-0.7044221	0.00E+00	0.00E+00	5.02	0.39	high	polr2a	1st exonic non-coding
cg05315670	BAD	20817948	-1.56E-01	4.28E-02	1.66E-02	9.06	0.03	high	polr2a	promoter
cg24310460	S100PBP; YARS	33146521	-0.3048714	1.15E-01	5.60E-02	9.32	0.21	low	polr2a	promoter
cg26342670	WIBG	114178495	-0.4354451	3.01E-05	1.20E-05	12.14	0.03	low	polr2a	promoter
cg13462232	DSTN	93544534	-3.14E-01	6.31E-10	2.44E-10	8.78	0.04	high	polr2a	promoter
cg06516445	DCUN1D5	38206839	-1.69E-01	2.81E-02	1.18E-02	10.44	0.01	high	polr2a	1st exonic non-coding
cg21509931	GPS2	19638859	-2.11E-01	1.95E-06	3.53E-07	8.63	0.03	high	polr2a	promoter
cg15790941	C4orf34	56119237	-1.89E-01	2.54E-03	3.96E-04	11.46	0.03	low	polr2a,nfya	1st exonic non-coding
cg13692001	EID2B	128842125	-3.43E-01	3.77E-05	1.31E-05	11.24	0.04	high	polr2a	promoter
cg05728201	YTHDC1	33055971	-0.77143	0.102778	0.02632	4.94	0.02	none	none	intronic
cg06914050	PSPH; CCT6A	94964516	-0.5062945	6.60E-03	8.45E-03	6.10	0.05	low	sp1,nfya	promoter
cg24398318	PPM1K	31926623	-1.91E-01	1.80E-05	2.91E-06	10.45	0.02	high	polr2a,e2f1	1st exonic non-coding
cg25335229	ZNF616	72874528	-0.2711361	1.15E-13	1.43E-14	9.79	0.08	high	polr2a	1stintronic
cg13537283	CCDC86	39715747	-2.79E-01	5.27E-08	6.87E-09	14.85	0.02	moderate	polr2a	promoter
cg19323951	SLC22A20	47755353	-2.46E-01	9.70E-04	1.13E-04	10.08	0.05	low	polr2a,cebpb	promoter
cg22903286	KIAA1737	121297486	-4.42E-01	3.76E-09	2.57E-09	2.01	0.02	moderate	polr2a	promoter
cg22137448	RNPS1	86974838	-2.51E-01	9.92E-04	7.31E-04	5.62	0.03	low	polr2a,nfya	promoter
cg22019999	WBP4	159436034	-2.14E-01	2.95E-05	5.67E-06	9.91	0.02	low	polr2a	promoter
cg23255774	ZNF460	17565885	-2.31E-01	7.37E-04	1.31E-04	NA	0.05	none	none	1st exonic non-coding
cg13227621	ZSWIM5	85843214	-0.2282865	4.92E-10	4.77E-11	10.35	0.01	low	polr2a	1st exonic non-coding
cg19679633	C5orf38; IRX2	5296031	-0.2716707	5.05E-08	9.10E-09	8.10	0.03	none	polr2a	1st exonic non-coding
cg11891983	RPL17; SNORD58A	45671361	-3.98E-01	3.58E-10	7.94E-11	10.22	0.07	none	none	1stintronic
cg26894438	ZSWIM7; TTC19	15902633	-0.3833345	0.00E+00	0.00E+00	8.03	0.03	high	polr2a	1st exonic non-coding
cg13595191	SNORD43; RPL3	39640517	-3.28E-01	1.06E-03	2.42E-04	8.62	0.02	low	polr2a	promoter

cg04406910	SKIV2L; RDBP	1619080	-2.34E-01	4.52E-06	9.92E-07	6.47	0.03	high	none	1stintronic
cg23623270	SLC25A39	228290140	-3.73E-01	0.00E+00	0.00E+00	8.53	0.03	none	ctcf	intronic
cg05367248	MCM10	63581631	-3.60E-01	7.71E-13	4.30E-13	8.98	0.06	none	none	intronic
cg16727585	ACACB	103570781	-1.81E-01	5.26E-02	1.96E-02	1.57	0.15	none	none	intronic
cg03899775	LARS2	52643207	-3.94E-01	5.19E-10	1.12E-10	9.55	0.04	moderate	polr2a,cebpb	promoter
cg02044989	PYGB	131591782	-2.33E-01	1.77E-03	1.95E-04	8.93	0.02	low	polr2a	1stintronic
cg25012434	PTPRK	35325348	-0.3106115	9.12E-03	2.50E-03	11.92	0.02	moderate	polr2a	1st exonic non-coding
cg22865402	MIR548F1	186344630	-0.3683788	9.03E-04	2.75E-04	9.69	0.02	high	polr2a,ctcf	promoter
cg16929354	SON; GART	57791959	-0.2303711	3.20E-02	3.44E-03	5.11	0.04	high	polr2a, ny3c	1st exonic non-coding
cg10088527	HTRA2; AUP1	37157995	-0.668515	4.21E-52	2.03E-52	5.36	0.28	none	none	intronic
cg18338863	MARCKS	60457901	-3.98E-01	2.02E-16	1.55E-16	10.75	0.03	low	none	promoter
cg12597276	SRFBP1	473007	-0.1829034	1.44E-02	3.01E-03	8.66	0.01	moderate	polr2a	1st exonic non-coding
cg25324601	NT5C2	53935265	-0.2742943	5.81E-14	7.41E-15	8.83	0.03	moderate	ctcf	1st exonic non-coding
cg23675362	XPR1	111497064	-0.2194251	1.86E-07	1.33E-08	7.44	0.08	high	polr2a	1stintronic
cg14312359	SARS2; MRPS12	2653240	-0.3804768	3.07E-04	7.78E-05	5.24	0.07	none	none	intergenic
